# The IFIT2–IFIT3 antiviral complex targets short 5’ untranslated regions on viral mRNAs for translation inhibition

**DOI:** 10.1038/s41564-025-02138-w

**Published:** 2025-10-15

**Authors:** Dustin R. Glasner, Candace Todd, Brian Cook, Agustina D’Urso, Shivani Khosla, Elena Estrada, Jaxon D. Wagner, Mason D. Bartels, Chuan-Tien Hung, Pierce Ford, Jordan Prych, Kathryn S. Hatch, Brian A. Yee, Kaori M. Ego, Qishan Liang, Sarah R. Holland, James Brett Case, Kevin D. Corbett, Michael S. Diamond, Benhur Lee, Gene W. Yeo, Mark A. Herzik, Eric L. Van Nostrand, Matthew D. Daugherty

**Affiliations:** 1 School of Biological Sciences, University of California, San Diego, CA USA; 2 Department of Chemistry and Biochemistry, University of California, San Diego, CA USA; 3 Therapeutic Innovation Center, Baylor College of Medicine, Houston, TX USA; 4 Verna and Marrs McLean Department of Biochemistry and Molecular Pharmacology, Baylor College of Medicine, Houston, TX USA; 5 Department of Microbiology, Icahn School of Medicine at Mount Sinai, New York, NY USA; 6 Department of Cellular and Molecular Medicine, University of California, San Diego, CA USA; 7 Sanford Stem Cell Institute and Stem Cell Program, UC San Diego, La Jolla, CA USA; 8 Institute for Genomic Medicine, UC San Diego, La Jolla, CA USA; 9 Department of Molecular Microbiology, Washington University School of Medicine, St Louis, MO USA; 10 Department of Medicine, Washington University School of Medicine, St Louis, MO USA; 11 Department of Pathology and Immunology, Washington University School of Medicine, St Louis, MO USA; 12 Sanford Laboratories for Innovative Medicines, La Jolla, CA USA

**Keywords:** Viral host response, Virus-host interactions, Interferons, Innate immunity, Viral infection

## Abstract

Recognition of foreign RNA is critical for the innate immune response to viruses. Interferon (IFN)-induced proteins with tetratricopeptide repeats (IFIT) 2 and 3 are highly upregulated following viral infection, but mechanistic insight into their antiviral role is lacking. Here we demonstrate that short 5’ untranslated regions (UTRs), a characteristic of many viral mRNAs, can serve as a molecular pattern for innate immune recognition via IFIT2 and IFIT3. Structure determination of the IFIT2–IFIT3 complex at 3.2 Å using cryo-EM reveals a domain-swapped heterodimer that is required for recognition of the viral mRNA 5’ end, translation inhibition and antiviral activity. Critically, viral or host 5’ UTR lengths less than 50 nucleotides are necessary and sufficient to enable translation inhibition by the IFIT2–IFIT3 complex. Accordingly, diverse viruses whose mRNAs contain short 5’ UTRs, such as vesicular stomatitis virus and parainfluenza virus 3, are sensitive to IFIT2–IFIT3-mediated antiviral activity. Our work thus reveals a pattern of antiviral nucleic acid immune recognition that takes advantage of the inherent constraints on viral genome size.

## Abstract (web summary)

Viruses generally have compact genomes, resulting in many viral mRNAs with short 5’ untranslated regions. An antiviral complex exploits this feature of viral mRNAs to selectively inhibit viral protein synthesis and repress viral replication.

## Main

Recognition of foreign RNA is a critical component of immune sensing during viral infection. Interferon (IFN) serves as the first line of defence against viral infection, and its induction is triggered by one of several mechanisms sensing ‘non-self’ RNA or DNA in host cells. IFN signalling induces expression of a number of proteins (for example, OAS, MDA5 and PKR), which themselves bind to double-stranded RNA (dsRNA) in the cytoplasm to amplify innate immune signalling and activation, promote RNA degradation, or inhibit messenger (m)RNA translation^1–5^. Analogously, dsDNA sensing in the cytoplasm by cGAS and other proteins is essential for activation of IFN responses^6–8^. Other RNAs can be recognized as foreign, including RNAs with an uncapped 5’ tri- or di-phosphate end by RIG-I^1,2,5^, mRNAs with high CG-dinucleotide content by zinc finger antiviral protein (ZAP/PARP13)^9^, and endosomal single-stranded RNA (ssRNA) by TLR7 and TLR8 (ref. ^10^). Notably, some of these patterns are also found on ‘self’ nucleic acids, including dsDNA and CG-dinucleotides. Despite this, these nucleic acid sensing proteins comprise a multifaceted barrier to infection by a wide range of viruses.

Among the many RNA-sensing innate immune proteins are the IFIT (IFN-induced proteins with tetratricopeptide repeats) proteins, coded by an evolutionarily dynamic family of genes that is highly upregulated during viral infection^11–13^. Notably, IFIT1 and IFIT1B proteins recognize ‘non-self’ methylation patterns on the 5’ cap structures of viral mRNAs, leading to inhibition of their translation^13–20^. In contrast, the role of IFIT2 and IFIT3 in the antiviral response is less clear. Several studies have implicated IFIT2 and IFIT3 in the restriction of viral infection in vitro and in vivo^11,21–25^, and human IFIT3 interacts with and potentiates the effects of human IFIT1 (ref. ^26^). In other studies, IFIT2 has been shown to have a proviral effect on influenza virus replication^27^ and activate apoptosis^28–30^, whereas IFIT3 may abrogate IFIT2-induced apoptosis^28,31^. While these studies indicate that IFIT2 and IFIT3 have important roles in innate immunity, the manner in which IFIT2 and IFIT3 exert direct antiviral effects has remained unclear.

In this study, we demonstrate that IFIT2 and IFIT3 are necessary and sufficient for antiviral activity against vesicular stomatitis virus (VSV) and parainfluenza virus 3 (PIV3). Using cryo-electron microscopy (cryo-EM), virological experiments and mRNA translation reporter assays, we demonstrate that IFIT2 and IFIT3 form a stable heterodimeric complex that facilitates recognition and translation inhibition of mRNA from several viruses. We determine that the molecular pattern that leads to IFIT2–IFIT3-mediated inhibition is the presence of short (<50 nucleotide (nt)) 5’ untranslated regions (5’ UTRs), which is a feature of mRNAs from many viral families. Our data elucidate a previously undescribed antiviral role for IFIT2 and IFIT3, and reveal that 5’ UTR length is a marker for non-self recognition of viral mRNAs during the innate immune antiviral response.

## Results

### IFIT2 and IFIT3 combine to exert potent antiviral effects in vitro

**Fig. 1:**
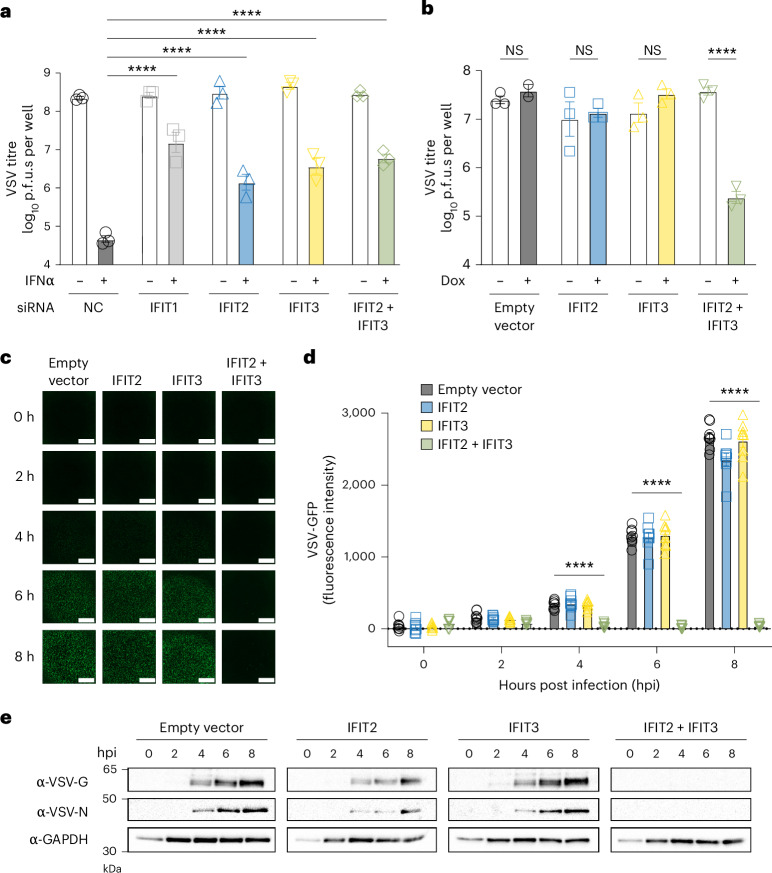
IFIT2 and IFIT3 act together to inhibit viral replication. **a**, Human A549 cells were treated with a non-targeting control siRNA (NC, black circles) as a negative control, or siRNA targeting IFIT1 (light grey squares), IFIT2 (blue triangles), IFIT3 (yellow triangles), or IFIT2 and IFIT3 in combination (green diamonds). In indicated conditions, cells were induced with IFNα (1,000 U ml^−1^). All cells were infected with VSV-GFP (0.01 MOI), and supernatant was collected at 16 hpi for titration (*n* = 3 biological replicates). p.f.u.s, plaque-forming units. **b**, Inducible Flp-In T-REx HEK293 cells expressing no IFIT (black circles), mouse IFIT2 (blue squares), mouse IFIT3 (yellow triangles), or mouse IFIT2 and IFIT3 together (green triangles) were mock treated or treated with doxycycline (500 ng μl^−1^) for 24 h and then infected with VSV-GFP (0.01 MOI). Supernatant was collected at 24 hpi for titration (*n* = 3 biological replicates). **c**–**e**, Using the same cell lines as in Fig. 1b, each cell line was treated with doxycycline (500 ng μl^−1^) for 24 h and infected with VSV-GFP (3.0 MOI). **c**, Images were taken at 0, 2, 4, 6 and 8 hpi. Scale bar, 1,000 μm. **d**, Images from experiments shown in **c** were processed and quantified for GFP fluorescence intensity (*n* = 8 biological replicates). **e**, Lysates from infected cells were collected at 0, 2, 4, 6 and 8 hpi and analysed by western blotting for expression of two viral proteins, VSV-G and VSV-N, and a loading control, GAPDH. All experiments were performed with 3 or more biological replicates with individual datapoints shown (**a**,**b**,**d**) or representative images shown (**c**). Data are represented as mean ± s.e.m. Statistical analyses: ordinary two-way analysis of variance (ANOVA) with Tukey’s post test and a single pooled variance (**a**,**d**); ****P < 0.0001; NS, not significant; ordinary two-way ANOVA with Šídák’s post test and a single pooled variance (**b**); ****P < 0.0001. Source data

**Extended Data Fig. 1:**
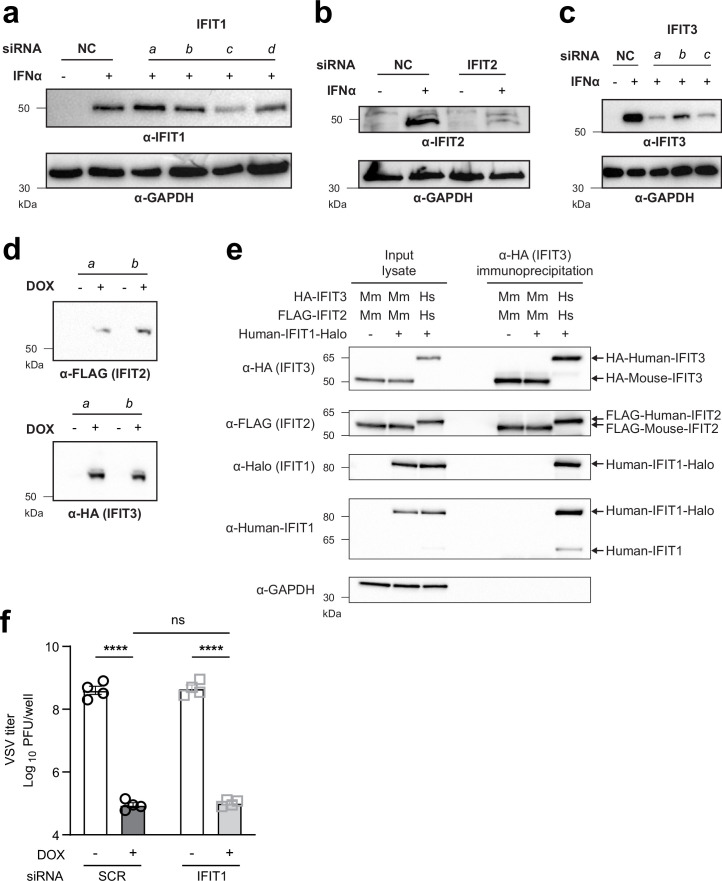
siRNA knockdown of IFITs in A549 cells and expression of IFIT2 and IFIT3 in FlpIn lines, related to Fig. 1. (**a-c**) A549 cells were treated with a non-targeting siRNA as a negative control or siRNA targeting human (**a**) *IFIT1*, (**b**) *IFIT2*, or (**c**) *IFIT3*. Twenty-four hours post-treatment, cells were harvested and subsequently processed and analyzed by western blotting. Representative images shown; 2 independent experiments. For downstream infection experiments, siRNA IFIT1-c and siRNA IFIT3-a were used. (**d**) We generated inducible Flp-In T-REx HEK293 cell lines co-expressing mouse IFIT2 and IFIT3. Cells were induced with 500 ng/mL DOX for 24 h before harvestting cell pellets for analysis by western blot. Representative images shown; 2 independent experiments. For all associated downstream experiments, Flp-In line *b* was used. (**e**) HEK293T were transfected with the indicated epitope-tagged plasmids expressing IFIT proteins from human (Hs) or mouse (Mm) and subjected to immunoprecipation with anti-HA conjugated beads to pull down HA-tagged human or mouse IFIT3. With pulldown of mouse IFIT3, we observe strong pulldown of mouse IFIT2, but no endogenous or overexpressed human IFIT1, which is consistent with previous literature^26^. In contrast, immunoprecipitation of human IFIT3 pulls down human IFIT2, and endogenous and overexpressed human IFIT1. Representative images shown; 2 independent experiments. (**f**) Inducible Flp-In T-REx HEK293 cells co-expressing mouse IFIT2 and IFIT3 were treated with a non-targeting control siRNA (NC, black circles) as a negative control or siRNA targeting IFIT1 (light grey squares). In indicated conditions, cells were mock treated or treated with doxycycline (500 ngμl^−1^) for 24 h and then infected with VSV-GFP (0.01 MOI), and supernatant was collected at 16 hpi for titration (n = 4 biological replicates). Statistical analyses: Data are represented as mean ± SEM. Ordinary two-way ANOVA with Tukey’s post-test and a single pooled variance. **** = p < 0.0001, ns = not significant. Source data

Studies with knockout mice have shown that IFIT2 and/or IFIT3 are required for antiviral activity against several RNA viruses, including VSV^11,22–25,32^. We first determined whether IFIT2 and IFIT3 contribute to the antiviral effects of type I IFN against VSV in human cells. Knockdown of either *Ifit1* (as a positive control^13,14,20^), *Ifit2*, or *Ifit3* in human A549 cells partially rescued viral replication following IFNα treatment (Fig. 1a and Extended Data Fig. 1a–c), suggesting that all three IFITs contribute to the antiviral effect of type I IFN.

Human IFIT1, IFIT2 and IFIT3 can interact with and regulate one another^14,26,33–35^, making it unclear from the above experiments whether IFIT2 and IFIT3 have antiviral activity distinct from IFIT1. We therefore took advantage of the observation that mouse IFIT3, unlike human IFIT3, does not interact with human IFIT1 (ref. ^26^) and generated HEK293 cell lines that express mouse IFIT2 and mouse IFIT3 alone or in combination under the inducible control of doxycycline (DOX) (Extended Data Fig. 1d). We confirmed that co-expression of mouse IFIT2 and IFIT3 do not immunoprecipitate human IFIT1, whereas co-expressed human IFIT2 and IFIT3 do (Extended Data Fig. 1e). Upon DOX induction, we observed no decrease in VSV titres when mouse IFIT2 or IFIT3 was expressed alone (Fig. 1b). However, when mouse IFIT2 and IFIT3 were co-expressed in HEK293 cells, we observed a >100-fold decrease in VSV titres (Fig. 1b), which was unchanged when we knocked down human IFIT1 (Extended Data Fig. 1f). Notably, a significant difference in GFP expression from the viral genome can be observed as early as 4 h post infection (hpi) (Fig. 1c,d). Together with our knockdown data, these results indicate that IFIT2 and IFIT3 are both necessary for effective IFN-mediated antiviral activity against VSV and sufficient to attenuate VSV replication when expressed in the absence of IFN stimulation.

We next evaluated the effect of mouse IFIT2 and IFIT3 expression on VSV-encoded protein levels during infection. At 0, 2, 4, 6 and 8 hpi, infected cells were collected and analysed by western blotting. Expression of IFIT2 and IFIT3 together, but not alone, prevented detectable accumulation of VSV-G and -N proteins compared to control cells (Fig. 1e). These data support a model in which IFIT2 and IFIT3 cooperate during the antiviral response to disrupt an early step of viral infection, resulting in lower viral protein expression.

### Structure of the mouse IFIT2–IFIT3 heterodimer reveals the basis for antiviral complex assembly

**Fig. 2:**
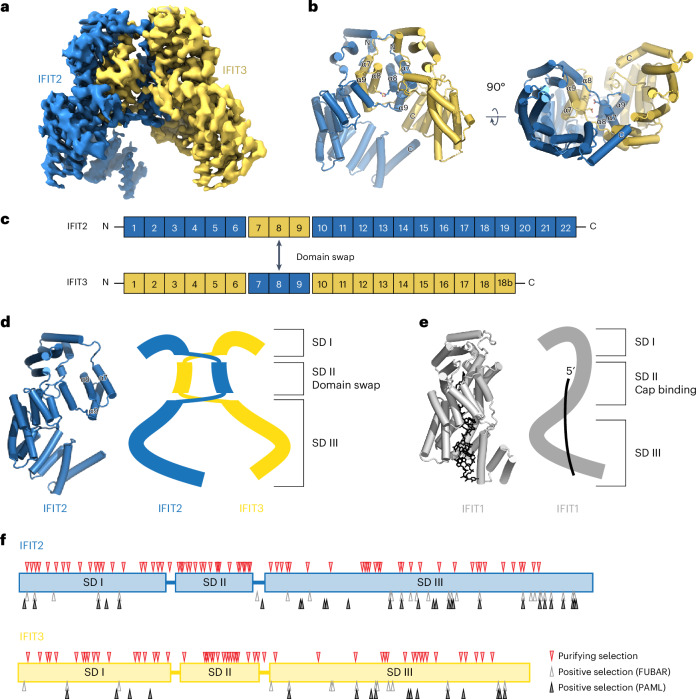
Structure of the IFIT2–IFIT3 heterodimer reveals a conserved interaction surface required for antiviral activity. **a**, Cryo-EM density map of the mouse IFIT2–IFIT3 heterodimer. **b**, Structural model of the IFIT2–IFIT3 heterodimer. **c**, Linear model of the IFIT2–FIT3 heterodimer, with helices indicated, including helices 7–9 that are involved in the domain swap. **d**, Isolated IFIT2 monomer from the structure (left) and schematic of the IFIT2–IFIT3 heterodimer (right), with subdomains (SDs) indicated. The SD II domain swap, which forms a continuous superhelix with SD I and SD III, is illustrated. **e**, Structure (left) and cartoon (right) of RNA-bound IFIT1 (PDB 6C6K (ref. ^26^), highlighting the difference in SD II orientation between IFIT1 and IFIT2/3 and the importance of SD II in IFIT1 for 5’ cap binding. **f**, Evolutionary analyses of IFIT2 and IFIT3 across >20 rodent species, revealing codons evolving under purifying selection (red triangles, from FUBAR analysis) and positive selection (grey and black triangles, from FUBAR and PAML analyses, respectively).

**Extended Data Fig. 2:**
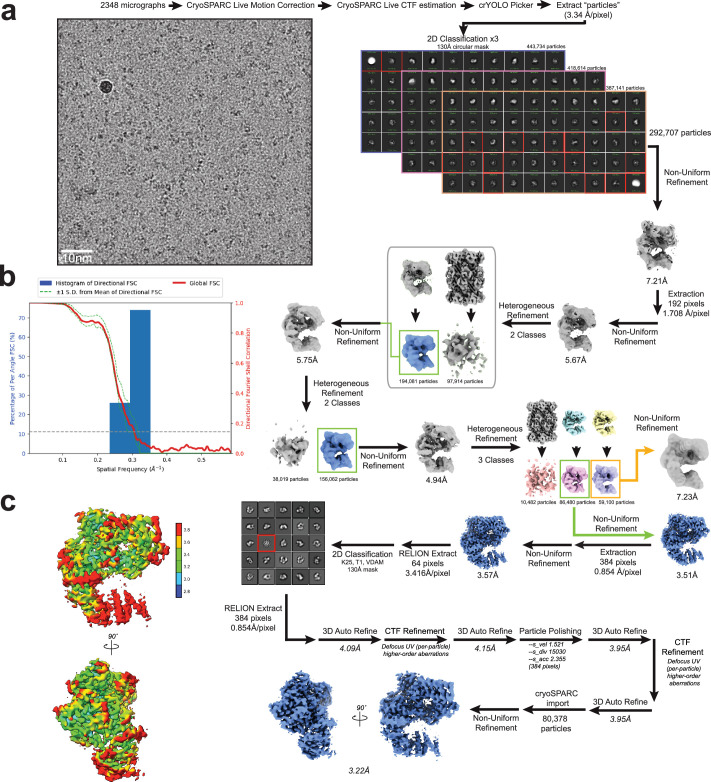
Cryo-EM data processing for IFIT2-IFIT3, related to Fig. 2. (**a**) Workflow for structure determination. See **Methods** for a detailed explanation of data processing. For 2D classes, classes outlined in red were excluded from later steps. For 3D classes, classes outlined in green were selected for later steps. The final refined map showed a resolution of 3.22 Å (gold-standard FSC criterion of 0.143). (**b**) Directional Fourier Shell Correlation analysis for the final map, calculated by 3DFSC^66^. (**c**) Two views of the final map, colored by local resolution (blue: 2.8 Å or better; red: 3.8 Å or worse).

**Extended Data Fig. 3:**
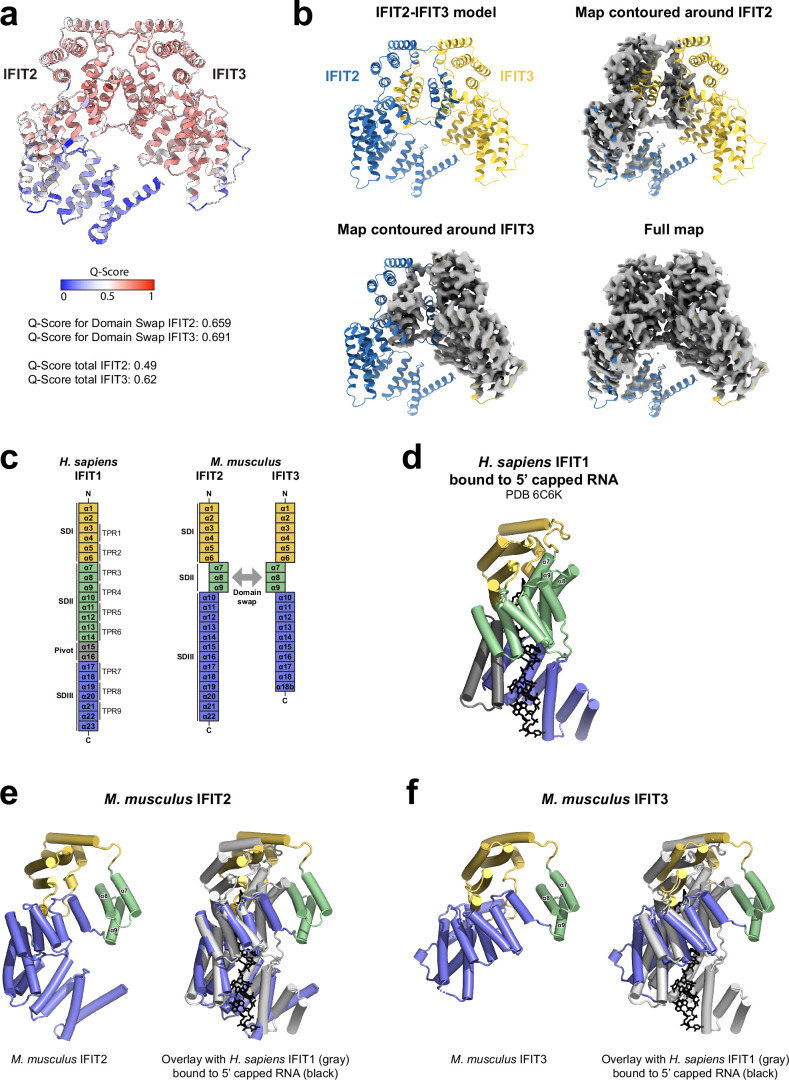
Comparison of IFIT structures, related to Fig. 2. (**a**) Model of the IFIT2-IFIT3 heterodimer, colored by Q score, which measures the agreement of each residue with the experimental map. (b) IFIT2-IFIT3 model colored by protomer with IFIT2 blue and IFIT3 yellow, and experimental cryoEM map contoured around the IFIT2 protomer (top left), contoured around the IFIT3 protomer (bottom right), or showing the full map (bottom right). (**c**) Linear representation of human IFIT1 and mouse IFIT2 and IFIT3 domains showing helix numbers and subdomains (SDs). (**d**) Structure of capped RNA-bound IFIT1 (PDB 6C6K ref. ^26^), colored by SD as in panel c. (**e**) (Left) Structure of mouse IFIT2 extracted from the IFIT2-IFIT3 heterodimer, colored by SD as in panel c. The green helices represent the domain-swapped helices during IFIT2-IFIT3 heterodimer formation. (Right) Mouse IFIT2 overlaid with IFIT1 (gray) bound to capped RNA (black). (**f**) Same as panel e, but with mouse IFIT3.

Although IFIT2 and IFIT3 are known to form a stable heterodimer^33^, the structural basis of IFIT2–IFIT3 complex formation had not been characterized. We therefore determined the structure of a 1:1 complex of mouse IFIT2 and IFIT3 to 3.2-Å resolution by cryo-EM (Supplementary Table 1 and Extended Data Fig. 2). IFIT proteins are composed of tandem α-helical tetratricopeptide repeats (TPR) that can fold into superhelical spiral structures^12,15,18,26,36,37^. In the case of monomeric IFIT1 and monomeric IFIT5, the superhelical structure is broken into four domains termed subdomain (SD) I, SD II, Pivot and SD III^15,18,26^. In our structure, each monomer of the mouse IFIT2–IFIT3 heterodimer also adopts an all-α-helical structure, with IFIT2 containing 22 α-helices and IFIT3 containing 19 α-helices (Fig. 2a–c and Extended Data Fig. 3a–c). In the assembled IFIT2–IFIT3 heterodimer, the two proteins are oriented parallel to one another and associate through a domain swap involving α-helices 7–9 in SD II (Fig. 2b–d and Extended Data Fig. 3a–c). The large, buried surface area that results from this domain swap (>4,000 Å^2^ per protomer) provides a molecular explanation for the stability of the IFIT2–IFIT3 complex^33^ and is similar to the domain-swapped topology of a previously published crystal structure of an IFIT2 homodimer^37^. Importantly, these changes in SD II orientation between IFIT1 and IFIT2–IFIT3 also alter the IFIT association with RNA. In RNA-bound IFIT1 (refs. ^15,18,26^), α-helices 7–9 in SD II form a clamp around the bound 5’ end of the mRNA, whereas in IFIT2–IFIT3, α-helices 7–9 are domain swapped, such that the equivalent deep clefts in this complex include structural elements from both IFIT2 and IFIT3 (Fig. 2d,e and Extended Data Fig. 3d–f). As a consequence, SD II is shorter in IFIT2 and IFIT3 relative to IFIT1 and IFIT5, and the α-helices C-terminal to SD II (α-helices 10–22 in IFIT2 and α-helices 10–19 in IFIT3) form a single continuous superhelix that comprises SD III (Fig. 2c,d and Extended Data Fig. 3c–f).

To investigate the importance of the domain-swapped SD II, we performed evolutionary analyses on IFIT2 and IFIT3 across species. Consistent with the importance of SD II for function, we observed strong signatures of conservation (purifying selection) for many residues in this region of the protein in both rodents and primates (Fig. 2f, Extended Data Table 1 and Supplementary Table 2). These evolutionary analyses also revealed strong signatures of positive selection acting on several residues in SD I and especially SD III of both IFIT2 and IFIT3 in rodents and primates (Fig. 2f, Extended Data Table 1 and Supplementary Table 2). Such signatures of recurrent positive selection are characteristic of host immunity proteins that are engaged in direct evolutionary arms races with viral antagonists^38–41^, and similarly strong signatures of positive selection have recently been described for IFIT1 (ref. ^20^). Our evolutionary analyses thus reveal not only the strong conservation of SD II, but also suggest that IFIT2 and IFIT3 are engaged in host–virus arms races as a result of their antiviral activities.

**Fig. 3:**
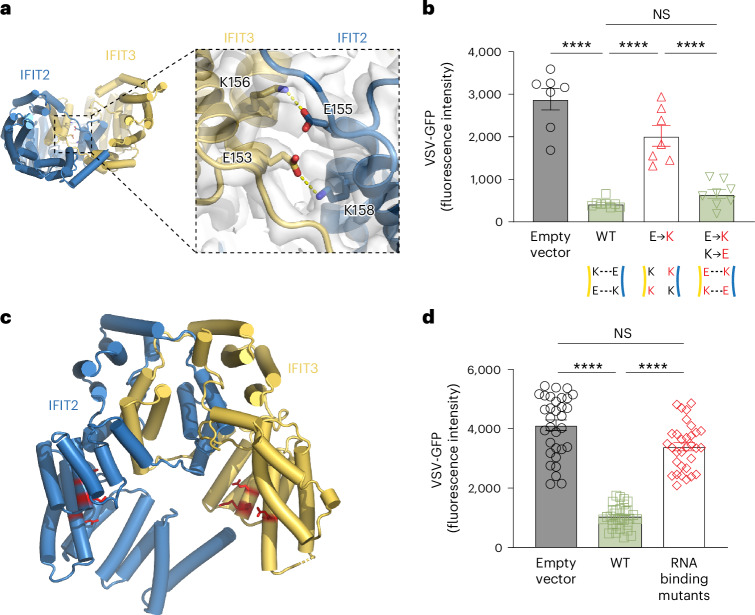
Structure-guided mutations of IFIT2 and IFIT3 abrogate antiviral activity. **a**, Zoomed-in view of the central helices in SD II that form a reciprocal salt bridge between IFIT2 (blue) and IFIT3 (yellow). Residue numbers are indicated. **b**, Importance of salt bridge residues for antiviral activity. HEK293T cells were transiently transfected with empty vector, wild-type (WT) IFIT2 and IFIT3, single E−>K mutant IFIT2 (E155K) and IFIT3 (E153K), or double mutant E−>K/K−>E IFIT2 (E155K K158E) and IFIT3 (E153K K156E). At 24 h post transfection, cells were infected with VSV-GFP (0.05 MOI), and fluorescence images were taken and quantified at 16 hpi (*n* = 7 biological replicates). **c**, Previously described RNA-binding residues^37,42^ (red) mapped on the IFIT2–IFIT3 heterodimer define a heterodimeric RNA-binding surface. **d**, RNA-binding mutants (mouse IFIT2: R250E, C253E, K254E, R287E; mouse IFIT3: Q249E, K252E, K253E, R286E) were tested for antiviral activity as described in **b** (*n* = 4 biological replicates, 32 technical replicates). In **b** and **d**, data are represented as mean ± s.e.m. and were analysed with an ordinary one-way ANOVA with Tukey’s multiple comparisons test. ****P < 0.0001. Source data

We next evaluated whether mutations at the heterodimer interface in SD II and the predicted RNA-binding surfaces in SD III would alter antiviral function. At the core of the SD II domain swap in the IFIT2–IFIT3 heterodimer is a pair of reciprocal glutamic acid–lysine (E–K) salt bridges that we hypothesized are important for complex formation and antiviral activity (Fig. 3a). As expected from introduction of a predicted K-to-K charge clash at the core of the heterodimeric interface, co-transfection of IFIT2 and IFIT3 single mutant (E−>K) constructs showed reduced antiviral activity against VSV-GFP compared with WT IFIT2 and IFIT3 (Fig. 3b). Importantly, when we reestablished the predicted salt bridges by introducing a second set of K−>E mutations, we restored the antiviral activity of IFIT2–IFIT3 (Fig. 3b). We also mutated a well-conserved set of residues in SD III on the basis of previously described mutations that were shown to disrupt mouse IFIT2 and human IFIT3 RNA binding^37,42^ (Fig. 3c). When cells were transfected with these mutant proteins and infected with VSV, we observed a dramatic reduction in antiviral activity relative to transfection with WT IFIT2–IFIT3 (Fig. 3d). These structural and functional data describe the basis for IFIT2–IFIT3 complex formation and its importance for antiviral activity.

### The IFIT2–IFIT3 antiviral complex binds VSV mRNAs near the start codons

Several IFITs have been described as having RNA-binding capabilities^13,18,19,33^. Accordingly, we assessed whether IFIT2 or IFIT3, alone or in combination, interacted with specific regions of VSV RNAs by performing enhanced UV-crosslinking and immunoprecipitation (eCLIP) on cells expressing mouse IFIT2 and/or IFIT3 and infected with VSV.

**Fig. 4:**
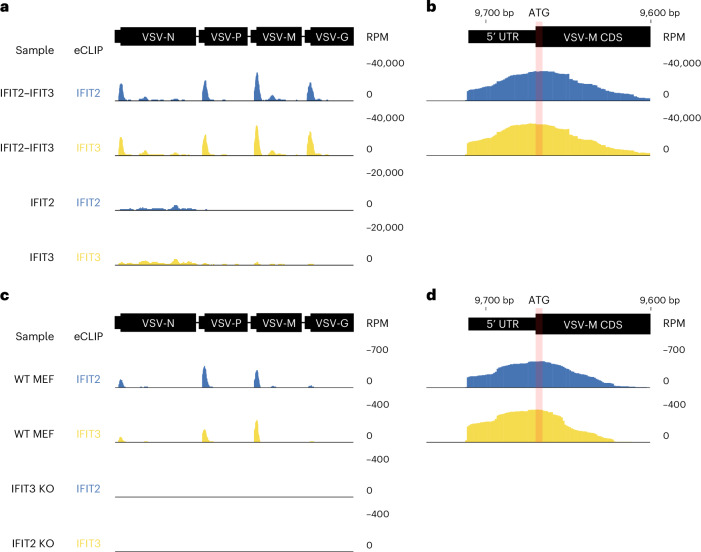
The IFIT2–IFIT3 heterodimer interacts with the 5’ end of VSV mRNAs near the start codons. **a**–**d**, Browser tracks of VSV reads uniquely mapped to the viral genome as captured by eCLIP of IFIT2 or IFIT3 in (**a**,**b**) IFIT-expressing Flp-In T-REx HEK293 lines and (**c**,**d**) wild-type and *Ifit2* or *Ifit3* knockout MEFs. eCLIP data are plotted as reads per million (RPM) mapped across the VSV genome normalized to the total RNA from that region of the viral genome. **b**,**d**, Zoomed-in browser tracks for 293 (**b**) and MEF (**d**) lines highlighting peaks of IFIT2–IFIT3 binding proximal to the start codon of the VSV-M gene.

**Extended Data Fig. 4:**
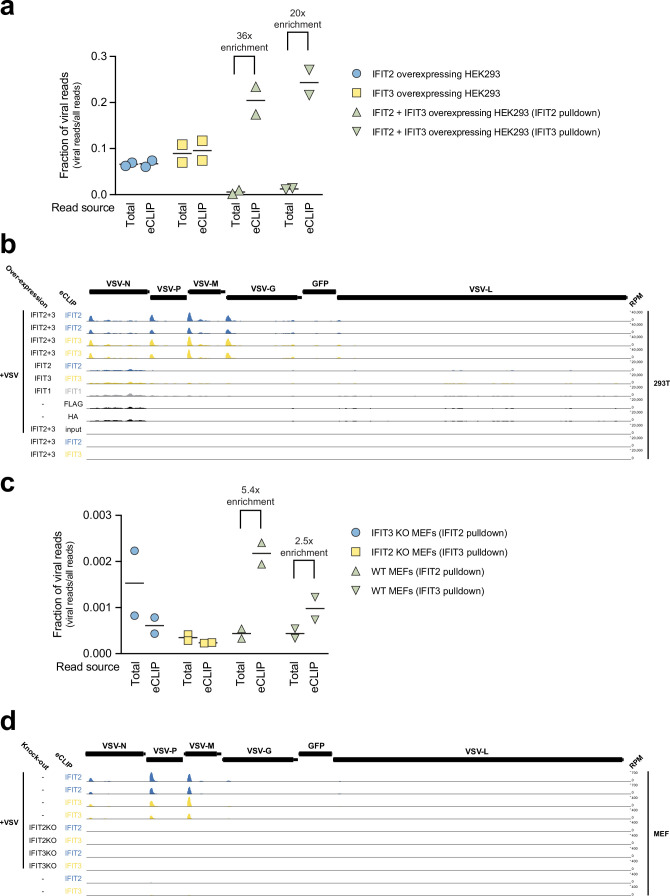
Additional eCLIP data, related to Fig. 4. (**a**) Enrichment of VSV viral RNA in eCLIP samples from HEK293 cells overexpressing IFIT2-IFIT3. Fraction of viral RNA relative to all RNA (host+viral) from RNAseq (Total) and eCLIP (eCLIP) samples for each indicated VSV infected cell line. Each replicate is shown as a point, with the line showing the average fraction of viral reads. For IFIT2-IFIT3 overexpression, the fold enrichment of IFIT-bound (eCLIP) fraction relative to fraction of viral reads in RNAseq (Total) is indicated. (**b**) Full VSV genome coverage for additional samples from the eCLIP experiment described in Fig. 4, including uninfected controls, for human IFIT-expressing Flp-In T-REx HEK293 lines. (**c**) Same as panel a, but for eCLIP samples from VSV infected mouse wild-type and *Ifit2* or *Ifit3* knockout MEFs. (**d**) Same as panel b, but for MEFs. (**b, d**) Values shown are normalized reads per million (RPM) out of all reads mapped to the VSV genome. Source data

We first performed experiments in HEK293 cells ectopically expressing mouse IFIT2 and/or IFIT3. When IFIT2 and IFIT3 were expressed together, we observed strong immunoprecipitation of RNA sequences near the 5’ end of the positive (protein-coding) strand of each VSV gene and a clear enrichment of viral mRNAs in the eCLIP dataset relative to total RNA (Fig. 4a and Extended Data Fig. 4a,b). The profile of enriched viral RNA was nearly identical for IFIT2 and IFIT3 when co-expressed, whereas we did not observe such enrichment when IFIT2 or IFIT3 was expressed alone. Moreover, we observed the strongest enrichment of sequences crosslinking to IFIT2 and IFIT3 in regions that overlap the 5’ UTR and start codons of the VSV mRNA transcripts (Fig. 4a,b). These data suggest that the IFIT2–IFIT3 heterodimer selectively associates with VSV transcripts at sequences proximal to the start codons.

To test whether IFIT2 and IFIT3 associate with the 5’ regions of viral mRNAs during an endogenous IFN response, we performed eCLIP experiments in mouse embryonic fibroblasts (MEFs) in which *Ifit2* or *Ifit3a* and *Ifit3b* had been knocked out (Fig. 4c,d and Extended Data Fig. 4c,d). In WT MEFs induced with IFN, IFIT2 and IFIT3 crosslinked with RNA sequences near the start codons of VSV mRNAs and showed an enrichment of viral mRNAs in the eCLIP dataset. In contrast, this association was lost when either *Ifit2* or *Ifit3a* and *Ifit3b* were knocked out. Although there are differences between our data with ectopically expressing cells and IFN-induced cells, we observe a similar overall pattern in IFIT2 and IFIT3 association with the 5’ ends of VSV mRNAs and a necessity for both IFIT2 and IFIT3 together during the antiviral response. These data further support the observation that the IFIT2–IFIT3 complex associates with viral mRNAs near their start codons during an innate immune response in infected cells.

### The IFIT2–IFIT3 complex inhibits translation of mRNAs with short viral 5’ UTRs

**Fig. 5:**
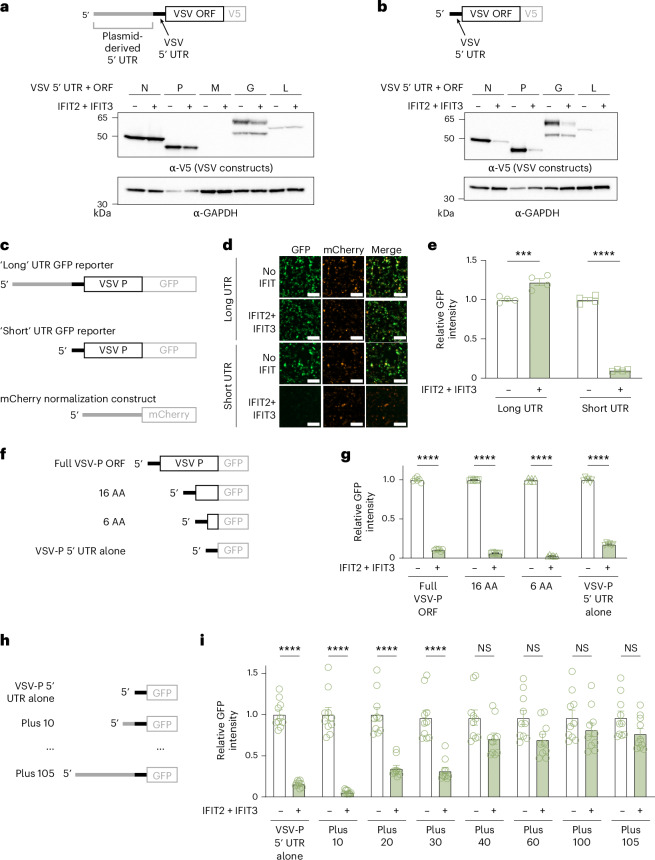
IFIT2–IFIT3 recognizes short viral 5’ UTRs. **a**, The 5’ UTR and ORF of each VSV gene was cloned into a mammalian expression plasmid fused to a C-terminal V5 tag (schematic at top). On the basis of 5’ RACE data (Extended Data Fig. 5a), we identified a 108-nt plasmid-derived extension (grey) appended to the 5’ end of the cloned VSV 5’ UTR (black). Each viral gene-expressing construct was co-transfected in the absence or presence of IFIT2–IFIT3 into HEK293T cells. At 24 h post transfection, cells were collected and lysates were analysed by western blotting. **b**, Following plasmid engineering, only 3 nt of plasmid-derived sequence remained at the 5’ end of each VSV UTR and ORF (schematic at top; Extended Data Fig. 5b). Transfections and western blotting were performed as in **a**. In **a** and **b**, experiments were performed independently at least twice. **c**, 5’ UTR and ORF schematics for fluorescence reporter constructs shown in **d** and **e**. **d**, HEK293T cells were transfected with the indicated GFP reporters in the absence or presence of co-transfected IFIT2–IFIT3. All wells were transfected with the control mCherry normalization construct. Images were taken at 24 h post transfection. Scale bars, 200 μm. **e**, Experiments were performed as in **d**. For each well (*n* = 4 biological replicates), 4 images were taken and the ratio of GFP:mCherry signal intensity (Extended Data Fig. 5d) was calculated for each image. All values were normalized to the average of the condition in which IFIT2–IFIT3 was not transfected. **f**, 5’ UTR and ORF schematics for GFP reporter constructs shown in **g**. AA, amino acids. **g**, Experiments with the indicated constructs were performed and quantified as in **e**. **h**, 5’ UTR and ORF schematics for GFP reporter constructs shown in **i**. Plasmid-derived sequence from the ‘long’ construct shown in **c** was added back with the indicated number of nt (for example, Plus 10). **i**, Experiments with the indicated constructs were performed and quantified as in **e** (*n* = 10 biological replicates). For **e**, **g** and **i**, data are represented as mean ± s.e.m. Statistical analyses: ordinary two-way ANOVA with Šídák’s post test and a single pooled variance. ****P < 0.0001, ***P = 0.0002. Source data

Our eCLIP data indicate that the IFIT2–IFIT3 complex interacts with mRNA sequences downstream of the 5’ cap (Fig. 4b,d). We therefore reasoned that viral genes may be sensitive to IFIT2–IFIT3-mediated inhibition even when expressed from a transfected plasmid. However, despite cloning the 5’ UTR and open reading frame (ORF) of VSV-N, -P, -M, -G and -L, which contain the sequences bound by IFIT2–IFIT3 in our eCLIP experiments, we did not observe changes in viral protein levels in the presence of IFIT2–IFIT3 expression (Fig. 5a).

**Extended Data Fig. 5:**
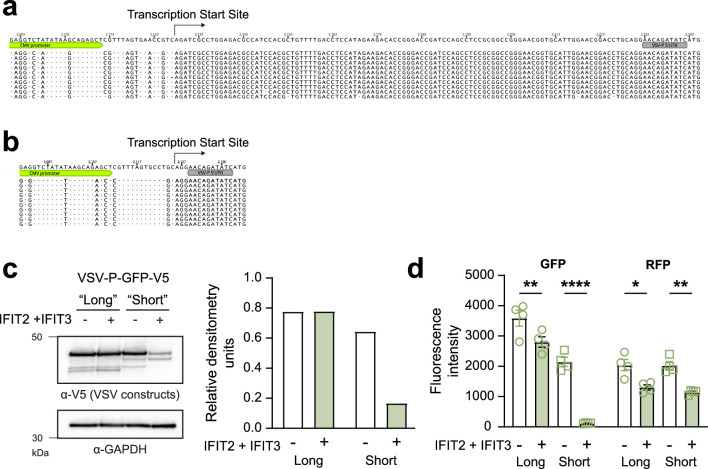
IFIT2-IFIT3 recognizes short viral 5’ UTRs, related to Fig. 5. (**a-b**) Plasmids expressing either a (**a**) long plasmid-derived 5’ UTR or (**b**) no plasmid-derived 5’ UTR immediately upstream of the VSV-P 5’ UTR were transfected into 293T cells. Twenty-four h post-transfection, cells were harvested, and RNA was isolated for 5’ RACE experiments. Ten clones from each construct were sequenced, and the transcription start site is marked for each plasmid. (**c**) Experiment was carried out as described in Fig. 5c, but instead of imaging, cells were harvested and lysates were analyzed by western blotting. Densitometry values were calculated using ImageJ. Experiment was performed independently twice. **(d)** Raw values from normalized data in Fig. 5d-e (n = 4 biological replicates). Statistical analyses: Data are represented as mean ± SEM. (**d**) Ordinary two-way ANOVA with Šídák’s multiple comparisons test and a single pooled variance. * = p < 0.05, ** = p < 0.01, **** = p < 0.0001. Source data

We next considered features of VSV mRNAs beyond their sequence that could differentiate them from most host mRNAs. One obvious difference is the vast discrepancy in 5’ UTR length between VSV mRNAs (10–41 nt) and human mRNAs (median length of 218 nt)^43^. We therefore hypothesized that 5’ UTR length might serve as a molecular pattern for viral mRNA recognition by IFIT2–IFIT3. Using 5’ rapid amplification of cDNA ends (RACE), we first determined that the transcription start site (TSS) of our plasmid was 105 nt upstream of where we cloned our viral sequences, resulting in a much longer 5’ UTR than natural VSV mRNAs (Fig. 5a and Extended Data Fig. 5a). Strikingly, when we removed the majority of this extraneous sequence (Fig. 5b and Extended Data Fig. 5b), we observed strong sensitivity of VSV-N, -P, -G and -L protein expression when IFIT2–IFIT3 was expressed (Fig. 5b). These data, which recapitulate the decreased protein expression during viral infection when IFIT2–IFIT3 is expressed (Fig. 1e), suggest that 5’ UTR length determines the sensitivity of an mRNA to IFIT2–IFIT3-mediated inhibition.

To facilitate more quantitative and high-throughput experiments, we established a fluorescence-based reporter assay. We first cloned GFP as a C-terminal fusion to VSV-P in our ‘long’ (105-nt plasmid-derived UTR followed by 10-nt VSV-P 5’ UTR) and ‘short’ (3 nt followed by 10-nt VSV-P 5’ UTR alone) plasmids (Fig. 5c). Consistent with our western blotting data (Fig. 5a,b and Extended Data Fig. 5c), GFP fluorescence signal was significantly decreased only when it was expressed with a ‘short’ 5’ UTR and IFIT2–IFIT3 was co-expressed (Fig. 5d,e and Extended Data Fig. 5d).

**Extended Data Fig. 6:**
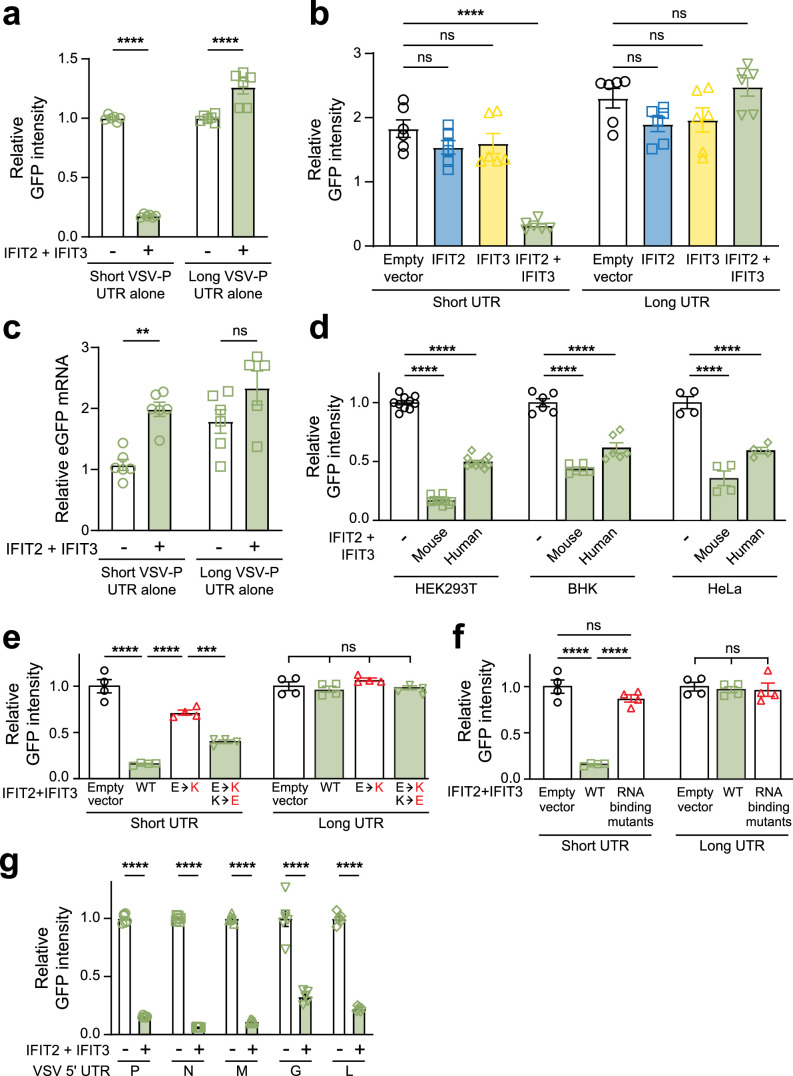
IFIT2-IFIT3 recognizes short viral 5’ UTRs, related to Fig. 5. (**a-b**) 293T cells were transfected with plasmids expressing the 10 nt VSV-P 5’ UTR and GFP with either a short or long plasmid-derived 5’ UTR upstream of the viral sequence, co-transfected in the absence or presence of (**a**) IFIT2-IFIT3 or (**b**) IFIT2 or IFIT3 alone. Experiments were performed as described in Fig. 5f-g. (**c**) Cells were transfected as in panel a, and 22 h post-transfection, cells were harvested, RNA was extracted, and RT-qPCR was conducted with GFP mRNA levels normalized to 18S RNA levels. Data are pooled from two independent experiments, and data are expressed as relative to the short VSV-P UTR alone, no IFIT2-IFIT3 condition. (**d**) 293T (left), BHK (middle), and H1-Hela (right) cells were transfected with plasmids expressing the 10 nt VSV-P 5’ UTR and GFP in the presence of empty vector (black circles), mouse IFIT2 and IFIT3 (green squares), or human IFIT2 and IFIT3 (orange diamonds). Experiments were performed as described in Fig. 5f-g. (**e-f**) 293T cells were transfected with plasmids expressing the 10 nt VSV-P 5’ UTR and GFP with either a short (left) or long plasmid-derived (right) 5’ UTR upstream of the viral sequence, co-transfected in the presence of empty vector (black circles), wild-type IFIT2-IFIT3 (green squares), (**e**) IFIT2-IFIT3 salt-bridge mutants (red triangles) and revertants (green triangles), or (**f**) IFIT2-IFIT3 RNA binding mutants (red triangles). Experiments were performed as described in Fig. 5g. (**g**) Cells were transfected with plasmids expressing GFP and the 5’ UTR of each VSV gene in the absence or presence of IFIT2-IFIT3. Experiments were performed as described in Fig. 5 g. Statistical analyses: Data are represented as mean ± SEM. (**a, b, c, g**) Ordinary two-way ANOVA with Šídák’s multiple comparisons test and a single pooled variance. ** = p < 0.01, *** = p < 0.001, **** = p < 0.0001, ns = not significant. (**d, e, f**) Ordinary one-way ANOVA with Tukey’s multiple comparison test and a single pooled variance. Comparisons are to the empty vector condition within each column. ns = not significant, *** = p < 0.001, **** = p < 0.0001. Source data

Using this reporter assay, we next determined that only the 10-nt VSV-P 5’ UTR, but not any part of the viral ORF, is required to sensitize GFP to repression by IFIT2–IFIT3 (Fig. 5f,g). Importantly, this sensitivity to IFIT2–IFIT3 expression was lost when the viral 5’ UTR was lengthened to include the original 105-nt plasmid-derived UTR (Extended Data Fig. 6a), and neither IFIT2 nor IFIT3 alone inhibited translation of the VSV-P 5’ UTR-alone construct (Extended Data Fig. 6b). We further confirmed that the observed inhibitory effect on protein expression from the short 5’ UTR was not due to IFIT-mediated changes in mRNA levels (Extended Data Fig. 6c) and confirmed that both human and mouse IFIT2–IFIT3 overexpression inhibited short 5’ UTR reporter output in three different cell lines (Extended Data Fig. 6d). We also demonstrated that mutations in the dimer swap interface and predicted RNA-binding surfaces, which reduced antiviral activity (Fig. 3), also selectively impact the short but not long 5’ UTR reporter (Extended Data Fig. 6e,f). Finally, we confirmed that the 5’ UTRs from VSV-N, -M, -G and -L, which range from 10–41 nt, are all sensitive in our reporter system (Extended Data Fig. 6g), demonstrating that multiple viral 5’ UTRs are susceptible to IFIT2–IFIT3-mediated mRNA translation inhibition.

**Extended Data Fig. 7:**
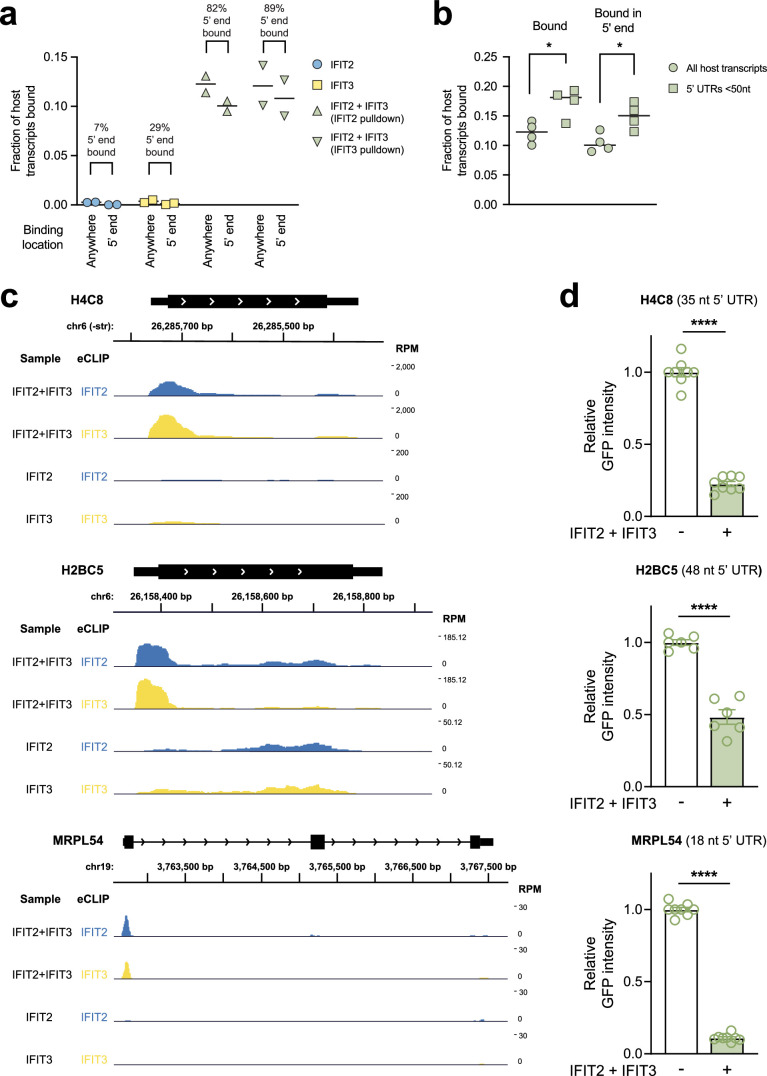
IFIT2-IFIT3 binds to host transcripts with short 5’ UTRs, which can sensitize a reporter to IFIT2-IFIT3-mediated inhibition, related to Fig. 5. (**a**) Fraction of all host transcripts (n ≥ 10,000 from RNAseq data) that eCLIP (greater than 3 in log10p and log2fc) with the indicated IFIT in VSV infected IFIT-overexpressing HEK293 cells. Transcripts with IFIT eCLIP signal in the 5’ end of the transcript are shown. Each replicate is shown as a point, with the average indicated by a line. (**b**) Fraction of all host transcripts (circles) or host transcripts with 5’ UTR lengths <50 nt ( ~ 11% of all host transcripts) (squares) that are bound in VSV infected IFIT2-IFIT3 overexpressing cells. As in panel a, transcripts with IFIT eCLIP signal in the 5’ end of the transcript are also shown. Individual replicates (combined from both IFIT2 and IFIT3 pulldowns) are shown as points, with the average value shown as a line. (**c**) Coverage of three short 5’ UTR-containing human genes (top: H4C8, 5’ UTR AGGUUUCUUAAGUUGGUUUUAGAAGUUGCUUAGUC; middle: H2BC5, 5’ UTR AUUAUUUUCUCAGGUGUUUGCAACAGUGUUCUAACUAUUAACGCUACG; bottom: MRPL54, 5’ UTR AAGCUGCCCGCAAUACGUC) from the eCLIP experiment described in Fig. 4a-b. Values shown are normalized reads per million (RPM) out of all reads mapped to the human genome. (**d**) HEK293T cells were transfected with GFP expression plasmids containing the indicated human 5’ UTRs from panel c in the absence or presence of co-transfected IFIT2-IFIT3. Experiments were performed as in Fig. 5e. Statistical analyses: Data are represented as mean ± SEM. (**b, d**) Ordinary two-way ANOVA with Šídák’s multiple comparisons test and a single pooled variance. * = p < 0.05, **** = p < 0.0001. Source data

Interestingly, in our eCLIP data, we also observed some host mRNAs that were bound in the 5’ UTR region by IFIT2–IFIT3 (Extended Data Fig. 7). As with viral RNAs, we observed that IFIT2 and IFIT3 expression alone did not lead to substantial host RNA binding, and that the majority of host transcripts (>80%) bound by IFIT2–IFIT3 showed eCLIP peaks in the 5’ end of the transcript (Extended Data Fig. 7a). Moreover, while the median human 5’ UTR is >200 nt, there was an enrichment for IFIT2–IFIT3 binding to host transcripts with short (<50 nt) 5’ UTRs, which were also predominantly bound in the 5’ end (Extended Data Fig. 7b). Analysis of three such transcripts, with 5’ UTRs ranging from 18–48 nt, showed clear eCLIP peaks at the 5’ end only when IFIT2–IFIT3 were co-expressed (Extended Data Fig. 7c) and these same host 5’ UTRs could sensitize GFP to inhibition by IFIT2–IFIT3 in our reporter system (Extended Data Fig. 7d). While the impact of IFIT2–IFIT3 on host mRNA translation remains to be fully explored, these data further support length-dependent inhibition by IFIT2–IFIT3.

The above data on viral and host transcripts show that 5’ UTRs below a certain length are sensitive to IFIT2–IFIT3 inhibition. To identify the 5’ UTR length threshold for IFIT2–IFIT3-mediated translation inhibition, we progressively added length to the sensitive 10-nt VSV-P 5’ UTR, increasing it back to the complete plasmid-derived 105 nt (Fig. 5h). Using this approach, we demonstrated that the strongest IFIT2–IFIT3-mediated translation inhibition occurs when the total length of the 5’ UTR is <50 nt (Fig. 5i).

### IFIT2–IFIT3 has broad antiviral activity driven by the length of 5’ UTRs

**Fig. 6:**
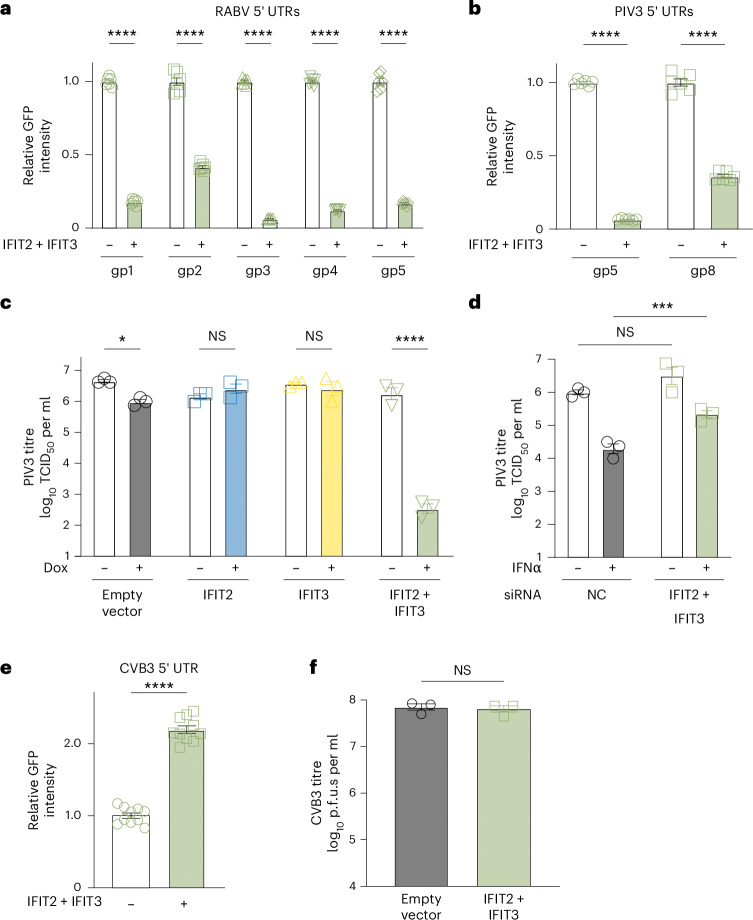
IFIT2–IFIT3 has broad antiviral activity driven by the length of 5’ UTRs. **a**,**b**, HEK293T cells were transfected with GFP expression plasmids containing the indicated viral 5’ UTRs from RABV (**a**) or PIV3 (**b**) in the absence or presence of co-transfected IFIT2–IFIT3. Experiments were performed as in Fig. 4e. **c**, Inducible Flp-In T-REx HEK293 cells expressing no IFIT (black circles), IFIT2 (blue squares), IFIT3 (yellow triangles), or IFIT2 and IFIT3 together (green triangles) were mock treated or treated with doxycycline (500 ng μl^−1^) for 24 h and then infected with PIV3-GFP (0.1 MOI). Supernatant was collected at 48 hpi and quantified using TCID_50_. **d**, A549 cells were treated with a non-targeting control siRNA (NC, black circles) as a negative control, or siRNA targeting *IFIT2* and *IFIT3* in combination (green diamonds). In indicated conditions, cells were induced with IFNα (1,000 U ml^−1^). All cells were infected with PIV3-GFP (0.1 MOI), and supernatant was collected at 48 hpi for quantification using TCID_50_. **e**, HEK293T cells were transfected with a GFP expression plasmid containing the 742-nt CVB3 5’ UTR in the absence or presence of co-transfected IFIT2–IFIT3. **f**, Inducible Flp-In T-REx HEK293 cells expressing no IFIT (black circles), IFIT2 (blue squares), IFIT3 (yellow triangles), or IFIT2 and IFIT3 together (green triangles) were mock treated or treated with doxycycline (500 ng μl^−1^) for 24 h and then infected with CVB3 (0.1 MOI). Supernatant was collected at 24 hpi and titred. In **a**–**f**, all experiments were performed with 3 or more biological replicates, with individual datapoints shown. Data are represented as mean ± s.e.m. Statistical analyses: ordinary two-way ANOVA with Šídák’s multiple comparisons test and a single pooled variance (**a**–**d**); *P < 0.05, ***P < 0.001, ****P < 0.0001; two-tailed unpaired parametric *t*-test (**e**,**f**); ****P < 0.0001. Source data

The data above suggest that a 5’ UTR that is <50 nt in length can serve as a molecular pattern that sensitizes an mRNA to an IFIT2–IFIT3 complex. On the basis of this idea, we hypothesized that mRNA from other viruses with short 5’ UTRs would be sensitive to IFIT2–IFIT3 antiviral activity. We first tested the 12–30-nt 5’ UTRs from rabies virus (RABV), which is in the same *Rhabdoviridae* family as VSV, and found that all RABV UTRs are sensitive to IFIT2–IFIT3-mediated inhibition (Fig. 6a). These data are consistent with observations that RABV is more pathogenic in mice in which either *Ifit2* or *Ifit3* is knocked out^21,23^.

**Extended Data Fig. 8:**
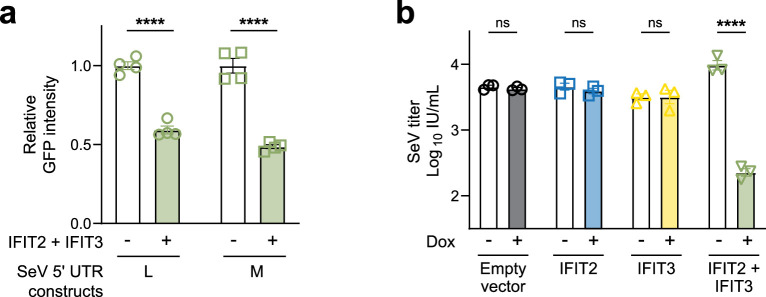
IFIT2-IFIT3 inhibits replication of the murine Sendai Virus, related to Fig. 6. (**a**) HEK293T cells were transfected with GFP expression plasmids containing the indicated 5’ UTRs from Sendai virus (SeV) in the absence or presence of co-transfected IFIT2-IFIT3. Experiments were performed as in Fig. 5e (n = 4 biological replicates). (**b**) Inducible Flp-In T-REx HEK293 cells expressing no IFIT (black circles), IFIT2 (blue squares), IFIT3 (yellow triangles), or IFIT2 and IFIT3 together (green triangles) were mock-treated or treated with doxycycline (500 ngμl^−1^) for 24 h and then infected with SeV-F1R-EGFP (0.1 MOI). Supernatant was collected at 42 hpi for titration (n = 3 biological replicates). Statistical analyses: Data are represented as mean ± SEM. (**a, b**) Ordinary two-way ANOVA with Šídák’s multiple comparisons test and a single pooled variance. **** = p < 0.0001, ns = not significant. Source data

We next tested another non-segmented negative-sense RNA virus with several short 5’ UTRs, the human parainfluenza virus 3 (PIV3). PIV3 encodes two mRNAs with 5’ UTRs <50 nt (gp5, 32 nt; gp8, 22 nt), both of which sensitize GFP to IFIT2–IFIT3-mediated inhibition in our reporter assay (Fig. 6b). As with VSV (Fig. 1b), neither IFIT2 nor IFIT3 alone exerted an antiviral effect on PIV3, but their co-expression significantly inhibited infection (Fig. 6c). Moreover, as with VSV (Fig. 1a), knockdown of *Ifit2* and *Ifit3* expression in human A549 cells blunted the antiviral effects of type I IFN on PIV3 replication (Fig. 6d). Finally, we tested Sendai virus (SeV), a rodent paramyxovirus previously shown to be susceptible to inhibition by IFIT2 in mice^44^. Similar to PIV3, SeV has several short 5’ UTRs that we found to be sensitive in our reporter assay (Extended Data Fig. 8a), and viral replication is sensitive to IFIT2–IFIT3 overexpression but not overexpression of either IFIT2 or IFIT3 alone (Extended Data Fig. 8b). Together, our data on VSV, RABV, PIV3 and SeV indicate that IFIT2–IFIT3 co-expression confers inhibitory activity against diverse viruses that encode mRNAs with 5’ UTRs shorter than 50 nt in length.

Finally, we evaluated whether a virus with a long 5’ UTR would be insensitive to the antiviral effects of IFIT2 and IFIT3. We cloned the single 742-nt 5’ UTR of coxsackievirus B3 (CVB3) upstream of GFP and tested it for sensitivity to IFIT2–IFIT3-mediated translation repression. Indeed, the CVB3 5’ UTR was insensitive to IFIT2–IFIT3 expression, with reporter levels actually increasing (Fig. 6e), suggesting that IFIT2–IFIT3 may be proviral in some contexts as previously shown^27^. Moreover, as predicted from our reporter assay data, ectopic expression of IFIT2 and IFIT3 did not inhibit CVB3 infection (Fig. 6f). These data suggest that viruses with long 5’ UTRs may evade recognition and translation inhibition by the IFIT2–IFIT3 complex.

## Discussion

Recognition of non-self viral mRNA is critical to controlling infection. Host cells must sense and distinguish viral mRNAs to prevent their translation and subsequent viral replication and formation of virions. Here we show that the interferon-stimulated genes (ISGs) IFIT2 and IFIT3 form a heterodimeric complex capable of exerting a potent antiviral effect against VSV (*Rhabdoviridae*) and both PIV3 and SeV (*Paramyxoviridae*), and that this activity is driven by recognition and translation inhibition of viral mRNAs containing short 5’ UTRs.

RNA viruses have an average genome length of ~9 kilobases^45^. With such limited genetic space, many viruses, especially small non-segmented negative strand RNA viruses, encode short 5’ UTRs, presumably to maximize nucleotides available for protein-coding sequences. Although RNA viruses have high rates of mutation and can undergo rapid change to genome sequences^46,47^, the length of viral 5’ UTRs is probably constrained due to limitations on overall genome size^46^, enabling IFIT2 and IFIT3 to have evolved recognition of this feature as a hallmark of RNA virus infection. Indeed, nearly every non-segmented negative-sense RNA virus that infects humans contains at least one mRNA with a 5’ UTR <50 nt, and our data with three of these viruses (PIV3, VSV and RABV) suggest that IFIT2–IFIT3 may be a broad restriction mechanism against them. However, our data revealing that the long 5’ UTR of CVB3 and CVB3 infection are insensitive to IFIT2–IFIT3 expression, indicate that evolving longer viral 5’ UTRs to mimic the length of host 5’ UTRs is one potential viral escape mechanism. Combined with studies showing that viruses can evolve RNA secondary structures, host-mimicking RNA methylation machinery and additional mechanisms to evade IFIT1 (refs. ^16,20^), these results suggest that IFITs may have had a profound effect on the evolution of the 5’ ends of viruses. In addition, viruses have probably developed additional strategies to evade translation inhibition by IFIT2–IFIT3. As has been shown with IFIT1 (ref. ^20^), we find that primate and rodent IFIT2 and IFIT3 have evolved under strong recurrent positive selection, especially in SD III. These evolutionary data mirror the strong signatures of positive selection described for other antiviral ISGs that are targeted by viral antagonists^16,20^ and indicate that some viruses that otherwise might be sensitive to IFIT2–IFIT3-mediated repression probably encode species-specific IFIT antagonists.

Intriguingly, we find that IFIT2–IFIT3 can also recognize host mRNAs with short 5’ UTRs and can inhibit translation of mRNAs containing host-derived short 5’ UTRs. These data suggest that, similar to many other antiviral effectors, IFIT2–IFIT3 may not perfectly distinguish viral from host mRNAs and therefore may have a role in host protein regulation. Indeed, several studies have indicated that IFIT2 and IFIT3 have host-regulatory functions^27–31^, although it is unknown whether any of those are due to inhibition of host mRNAs with short 5’ UTRs. Future research focusing specifically on host mRNA translation will need to be conducted to understand the consequences of IFIT2–IFIT3-mediated inhibition of host mRNA translation.

The precise mechanism by which the IFIT2–IFIT3 complex selectively inhibits translation of mRNAs with short 5’ UTRs also requires further study. Previous models based on ribosome structures and functional data have described a ‘blind spot’ in the 5’ 40–50 nt of human mRNAs, where efficient translation initiation occurs at the first start codon downstream of the blind spot^48^. However, viruses with short 5’ UTR-containing mRNAs are able to efficiently translate their proteins during infection. This paradox suggests that there are exceptions to the blind spot model and that viruses that express mRNAs with short 5’ UTRs probably co-opt non-canonical translational pathways. Indeed, VSV translation initiation is strongly dependent on the large ribosomal subunit protein, RPL40 (eL40), whereas bulk cellular translation is not^49^. Likewise, host mRNAs with short 5’ UTRs can contain a specific sequence known as a TISU (translation initiator of short 5’ UTR)^50,51^, and rely on specific ribosomal proteins and initiation factors for efficient translation^52,53^. The differential requirements for efficient translation of mRNAs with short 5’ UTRs and the similarity of our <50-nt length cut-off for IFIT sensitivity to the 40–50-nt ribosome blind spot lead us to hypothesize that IFIT2 and IFIT3 selectively inhibit a specialized translation pathway required for short 5’ UTRs, although further work will be required to understand this mechanism in depth.

In summary, our study identifies short mRNA 5’ UTRs as a molecular pattern that mammalian hosts can use to selectively inhibit viral replication. This pattern is present in a wide range of viral mRNAs and is sufficient to sensitize an mRNA to translation inhibition by the IFIT2–IFIT3 antiviral complex. Thus, 5’ UTR length is another point of conflict in the battle between viruses and hosts for mRNA translation control.

## Methods

The research described in this paper complies with all relevant ethics regulations and was approved by UCSD Environment, Health and Safety (EH&S) under Biohazard Use Authorization No. 2215. Animal experiments were carried out in accordance with the recommendations in the Guide for the Care and Use of Laboratory Animals of the National Institutes of Health. The protocols were approved by the Institutional Animal Care and Use Committee at the Washington University School of Medicine (Assurance number A3381-01).

### Cell culture and transient transfection

HEK293T cells, BHK-21 [C-13] cells and H1-HeLa cells were obtained from ATCC (CRL-3216, CCL-10 and CRL-1958, respectively) and grown in complete medium containing DMEM medum (Gibco), 10% FBS, 100 U ml^−1^ penicillin and 100 μg ml^−1^ streptomycin (Gibco). For transient transfections, HEK293T, BHK-21 or H1-HeLa cells were seeded the day before transfection in a 24-well plate (Genesee) with 500 μl of complete media. Cells were transiently transfected with 500 ng of total DNA and 1.5 μl of TransIT-X2 (Mirus Bio) following manufacturer protocol.

For generation of inducible cell lines, sequences for mCherry, IFIT2, IFIT3, or IFIT2 and IFIT3 separated by a P2A site were cloned into the Flp-In vector pcDNA5/FRT/TO. Flp-In T-REx HEK293 cells (Invitrogen, R78007) maintained in 5 μg ml^−1^ of blasticidin were transfected at 70% confluency with mCherry or IFIT constructs and the vector containing the Flp recombinase pOG44 in a 1:10 molar ratio using TransIT-X2 (Mirus Bio). After 1 day, cells were transferred to new dishes, and on the following day, hygromycin (100 μg ml^−1^) was added to cells. Following selection, cells were maintained in 5 μg ml^−1^ of blasticidin and 100 μg ml^−1^ of hygromycin. For induction of mCherry or IFIT proteins, cells were treated with 500 ng ml^−1^ of doxycycline for 24 h.

MEFs from *Ifit2*^−*/*−11^ and Δ*Ifit3a/b* mice were prepared from day 13.5–14.5 embryos according to published protocols^54^. Isolated MEFs were maintained in DMEM supplemented with 10% heat-inactivated fetal bovine serum (FBS) (Cytiva), 100 U ml^−1^ penicillin–streptomycin (Invitrogen), non-essential amino acids (Cellgro) and Glutamax (Gibco). Passage 0 (P0 MEFs) were frozen or split 1:4 when ~80% confluent (~3 days). To generate transformed MEFs, 5 × 10^6^ P1 primary MEFs were transfected with 10 μg of a plasmid (SV2)^55^ encoding the SV40 T antigen under control of a CMV promoter, using FuGene reagent (Promega) (3:1 μl FuGene to μg DNA ratio). Upon achieving confluence, MEF cultures were split 1:10. This process was repeated for ~10 passages, at which time the transformed MEFs were frozen or used for experiments.

### IFIT small interfering (si)RNA knockdowns

Specific siRNAs against *ifit1* (Integrated DNA Technologies; 5’-UAGACGAACCCAAGGAGGCUCAAGCUU-3’), *ifit2* (Horizon Discovery, M-012582-01-0050) and *ifit3* (Integrated DNA Technologies, TriFECTa RNAi kit - hs.Ri.IFIT3.13.1) were obtained from their respective manufacturers. A549 cells were seeded into 24-well plates. At 24 h after seeding, cells were transfected with 20 pmol of siRNA in Lipofectamine 2000 Transfection Reagent (Invitrogen) and allowed to incubate for 24 h before being used in subsequent infection experiments or being collected for western blot to validate knockdown efficiency.

### Viral stocks and infections

VSV-GFP^56^ was propagated in BHK cells. For siRNA experiments, siRNA-treated A549 cells in 24-well plates were induced with 500 U ml^−1^ of IFNα for 24 h. Cells were then infected at a multiplicity of infection (MOI) of 0.01 for 16 h before collection, and virus was quantified by plaque assay. For ectopic overexpression experiments, Flp-In T-REx HEK293 cells in 24-well plates were induced with doxycycline for 24 h. Cells were then infected at an MOI of 0.01 for 16 h before collection, and virus was quantified by plaque assay. For high-MOI experiments, Flp-In 293 cells in 96-well (imaging) or 24-well (western blotting) plates were infected at an MOI of 3.0, and cells were collected or plates were imaged at 0, 2-, 4-, 6- and 8 h post infection. For evaluation of structure-guided mutations, 293T cells cultured in 24-well plates were transfected at 6 h post seeding with IFIT constructs. At 18 h post transfection, cells were infected at an MOI of 0.05 for 16 h before imaging.

For siRNA experiments with PIV3-GFP and SeV-F1R-GFP^57^, siRNA-treated A549 cells in 24-well plates were induced with 500 U ml^−1^ of IFNα for 24 h. Cells were then infected at an MOI of 0.01, and supernatant was collected at 40 hpi. Virus was quantified using 50% tissue culture infectious dose (TCID_50_) analysis. For ectopic expression experiments, Flp-In T-REx HEK293 cells in 24-well plates were induced with doxycycline for 24 h before being infected at an MOI of 0.1 for 48 h (PIV3) or 42 h (SeV). Supernatant was then collected, and virus was quantified using TCID_50_.

CVB3 stocks were generated by co-transfection of CVB3-Nancy infectious clone plasmids with a plasmid expressing T7 RNA polymerase as previously described^58^. For ectopic overexpression experiments, Flp-In T-REx HEK293 cells in 24-well plates were induced with doxycycline for 24 h before being infected at an MOI of 0.1. Supernatant was collected at 40 hpi and quantified by plaque assay.

### Western blotting and antibodies

At 24 h post transfection, cells were resuspended in supernatant and collected, followed by centrifugation for 5 min at 587 × *g*. Cell pellets were washed with 1× PBS and lysed with 1× Bolt LDS sample buffer (Life Technologies) containing 5% β-mercaptoethanol at 98 °C for 7 min. The lysed samples were centrifuged at 21,130 × *g* for 2 min, followed by loading into 4–12% Bolt Bis-Tris Plus Mini Protein Gels (Life Technologies) with 1× Bolt MOPS SDS Running Buffer (Life Technologies). Following electrophoresis, gels were wet transferred onto nitrocellulose membranes using a Mini Blot Module (Life Technologies). Membranes were blocked with PBS-T containing 5% bovine serum albumin (BSA) (Spectrum), followed by incubation with primary antibodies (1:1,000) for VSV-G [8G5F11] and VSV-N [10G4] (Kerafast), V5 [D3H8Q] (Cell Signaling Technology), HA [3F10] (Roche), FLAG [M2] (Sigma), HaloTag (Promega), human IFIT1 [3G8] (Novus Biologicals) or GAPDH [14C10] (Cell Signaling Technology). Membranes were rinsed three times in PBS-T and then incubated with the appropriate HRP-conjugated secondary antibodies (1:10,000; goat anti-rabbit IgG, Bio-Rad; goat anti-mouse IgG, Bio-Rad; goat anti-rat IgG, Invitrogen). Membranes were rinsed again three times in PBS-T and developed with SuperSignal West Pico PLUS Chemiluminescent Substrate (Thermo Fisher). Blots were imaged on a Bio-Rad ChemiDoc MP using the Bio-Rad Image Lab Software suite (v.6.1.0).

### Immunoprecipitations (IPs)

HEK293T cells were seeded in 6-well plates and transfected the next day with plasmids expressing the indicated IFITs (600 ng each). Cells were collected in PBS at 24 h post transfection and centrifuged at 8,500 × *g* for 5 min at room temperature. Cell pellets were then flash frozen in liquid nitrogen and stored at −80 °C. For immunoprecipitation, cells were thawed on ice and incubated for 15 min in 500 μl of lysis buffer [20 mM Tris-Cl (pH 8.0), 150 mM NaCl, 15 mM MgCl_2_, 1% (v/v) Triton X-100, 1× Protease Inhibitor Mini Tablets (Thermo Scientific), 1 mM dithiothreitol and 4 U TURBO DNase (Invitrogen)]. Lysates were centrifuged at 500 × *g* for 5 min at 4 °C to pellet cell debris, and 440 μl of the supernatant was transferred to LoBind tubes (Eppendorf SE), while 50 μl of this supernatant was set aside to serve as ‘Input lysate’ control samples for immunoblots. Monoclonal anti-HA agarose beads (Sigma-Aldrich) were washed three times in 1 ml of lysis buffer, followed by centrifugation at 800 × *g* for 5 min at 4 °C and removal of the supernatant. A volume of 40 μl of a 1:1 agarose beads:lysis buffer mix was added to each LoBind tube containing lysate, followed by incubation on a rotator for 3.5 h at 4 °C. After incubation, samples were washed three times by centrifugation at 800 × *g* for 5 min 4 °C, discarding the supernatant and adding 1 ml of lysis buffer. After the final wash, the supernatant was discarded. To remove bound proteins from agarose beads, 100 μl of 2× LDS sample buffer (Life Technologies) containing 10% (v/v) 2-mercaptoethanol (Thermo Scientific) was added to the agarose beads. The same amount (100 μl) of 2× LDS sample buffer was added to input lysate control samples. Samples were heated at 98 °C for 7 min and centrifuged at maximum speed for 5 min. Samples were then loaded onto a 4–12% Bis-Tris gel (Invitrogen) and electrophoresed in 1× MOPS SDS running buffer (Invitrogen). Immunoblots for HA, FLAG, HaloTag, IFIT1 and GAPDH were performed as described above.

### Plasmids, constructs and molecular cloning

The coding sequences of mouse IFIT2 (NCBI accession BC050835) and mouse IFIT3 (NCBI accession BC089563) were cloned separately into the pcDNA5/FRT/TO backbone (Invitrogen) with an N-terminal 3×FLAG tag or the pQCXIP backbone (Takara Bio) with an N-terminal HA tag, respectively, and both were cloned into the pcDNA5/FRT/TO backbone (an N-terminal HA tag, followed by IFIT3, a P2A site, a 3×FLAG tag and IFIT2). IFIT2 and IFIT3 point mutants were generated using overlapping stitch PCR and cloned into their respective backbones. The 5’ UTR and coding sequence for each VSV gene (GenBank accession number NC_038236.1) was cloned into the pQCXIP backbone with a C-terminal V5 tag. Following 5’ RACE, ‘short’ 5’ UTR constructs were generated by truncating the pQCXIP 5’ UTR sequence. The ‘long’ and ‘short’ backbone constructs expressing VSV-P-V5 were used to further clone the VSV-P fluorescence reporter plasmids by subcloning GFP in between the VSV-P 5’ UTR and V5 tag. The mCherry normalization construct was generated by subcloning mCherry and a C-terminal 3×FLAG tag into the pcDNA5/FRT/TO backbone. All additional reporter constructs (including VSV-P truncations, UTR length constructs and viral 5’ UTRs [RABV (NCBI accession NC_001542)]; [PIV3 (NCBI accession NC_001796)]; [CVB3 (NCBI accession NC_038307)]; [SeV (NC_075392.1)]) were cloned using primers and inserted into the ‘short’ UTR reporter backbone upstream of GFP-V5. All generated plasmids were sequenced across the entire inserted region to verify that no mutations were introduced during the cloning process. Plasmids and primers used in this study can be found in Supplementary Table 3. Gene fragments were ordered from Twist Bioscience or Genscript. All newly created plasmids will be made available upon request.

### Protein expression and purification

For expression of the IFIT2–IFIT3 complex, we cloned codon-optimized genes encoding *M. musculus Ifit2 and Ifit3* into separate plasmid vectors for expression in *E. coli*, with *Ifit2* cloned into UC Berkeley Macrolab vector 2-BT (Addgene, 29666; ampicillin resistant) encoding a TEV protease-cleavable His6-tag, and *Ifit3* cloned into UC Berkeley Macrolab vector 13S-A (Addgene, 48323; spectinomycin resistant) with no tag.

Plasmids were co-transformed into *E. coli* Rosetta pLysS cells (EMD Millipore) and grown overnight at 37 °C in LB plus carbenicillin and spectinomycin. Saturated overnight cultures were used to inoculate six 1-l cultures of 2XYT media plus carbenicillin and spectinomycin, and cultures were grown at 37 °C with shaking at 180 r.p.m. to an optical density at 600 nm (OD_600_) of 0.8. Protein expression was induced by the addition of 0.25 mM IPTG, then cultures were shifted to 20 °C and grown another 16 h with shaking. Cells were collected by centrifugation and resuspended in nickel wash buffer (20 mM Tris-HCl pH 7.5, 500 mM NaCl, 20 mM imidazole pH 8.0, 2 mM beta-mercaptoethanol and 10% glycerol).

For protein purification, resuspended cells were lysed by sonication (Branson Sonifier), then cell debris was removed by centrifugation at 17,013 × *g* in a JA-17 rotor in an Avanti J-E centrifuge (Beckman Coulter) for 30 min. Clarified lysate was passed over a nickel column (5 ml HisTrap HP, Cytiva) in nickel wash buffer, then bound protein was eluted with nickel elution buffer (20 mM Tris-HCl pH 7.5, 75 mM NaCl, 250 mM imidazole pH 8.0, 2 mM beta-mercaptoethanol and 10% glycerol). Eluted protein was concentrated and buffer exchanged into nickel elution buffer containing 20 mM imidazole (Amicon Ultra, EMD Millipore), and the His_6_-tag on IFIT2 was cleaved by addition of 1:10 w/w ratio of purified TEV protease (S219V mutant, purified in-house from expression vector pRK793; AddGene, 8827)^59^, followed by incubation at 4 °C for 48 h. The reaction mixture was passed over a nickel column to remove cleaved His_6_-tags, uncleaved IFIT2 and His_6_-tagged TEV protease. The flow-through was concentrated, then passed over a size exclusion column (Superdex 200 Increase, Cytiva) in size exclusion buffer (20 mM Tris-HCl pH 7.5, 150 mM NaCl and 1 mM dithiothreitol), and fractions containing both proteins were pooled and concentrated.

### Cryo-EM grid preparation

Before use, UltrAuFoil 1.2/1.3 300 mesh grids were plasma cleaned for 12 s using a Solarus II plasma cleaner (Gatan). Purified IFIT2–IFIT3 at 3 mg ml^−1^ was applied to the grid in a 3-μl drop within the environmental chamber adjusted to 4 °C temperature and ~95% humidity in a Vitrobot Mark IV system (Thermo Fisher). After a 4-s incubation, the grids were blotted with a blot force of 4 for 4 s; the sample was then plunged frozen into liquid nitrogen-cooled liquid ethane.

### Cryo-EM data acquisition and image processing

All data were acquired at the UCSD Cryo-EM Facility on a Titan Krios G3 electron microscope (Thermo Fisher) operating at 300 kV and equipped with a Gatan BioContinuum energy filter. All images were collected at a nominal magnification of ×165,000 in EF-TEM mode (with a calibrated pixel size of 0.854 Å) on a Gatan K2 detector using a 20-eV slit width and a cumulative electron exposure of ~65 electrons Å^−2^ over 50 frames (Supplementary Table 1). Data were collected automatically using EPU (Thermo Fisher) with aberration-free image shift with a defocus range of −1 to −2.5 µm. Data collection was monitored live using the cryoSPARC Live platform (Structura Bio)^60^ where movies were patch motion corrected and patch contrast transfer function (CTF) estimated on the fly. Micrographs with a CTF estimation worse than 7 Å and/or a cumulative motion of more than 150 pixels were discarded.

An initial 484,841 particle picks were obtained with crYOLO^61^ picker using a general model, and these picks were imported into cryoSPARC^60^. Particles were extracted with a box size of 384 pixels and Fourier cropped to 96 pixels at 3.34 Å per pixel. These particles were subjected to three rounds of two-dimensional (2D) classification, where classes with proteinaceous features were chosen to move forward. The selected particles were subjected to an ab initio reconstruction to generate a starting model and carried forward to a non-uniform (NU) refinement using C1 symmetry. These particles were then re-extracted at a box size of 384 pixels with a Fourier crop to 128 pixels (1.708 Å per pixel), and a second NU refinement was performed. The particle stack was then subjected two rounds of a 2-class heterogeneous refinement, with one volume being the volume from the previous NU refinement and the other volume from EMD-4877 (20S proteasome)^62^, followed by NU refinement. In each round, the particles that contributed to the best volume that resembled the IFIT2–IFIT3 dimer were selected. A final 3-class heterogenous refinement was performed using two IFIT2–IFIT3 volumes and a 20S proteasome volume. The particles associated with the volume that showed the best secondary structure features was selected and NU refinement was performed, resulting in a 3.51-Å-resolution map. These particles were then re-extracted at a box size of 384 pixels with no Fourier cropping (0.854 Å per pixel) and then NU refined, resulting in a 3.57-Å-resolution map. Due to heterogeneity that was observed in the map, particles were then exported from cryoSPARC into RELION^63^, where they were extracted at a box size of 256 with a Fourier crop of 64 (3.34 Å per pixel). These particles were subjected to a round of 2D classification in which obvious junk classes were discarded. Selected particles were then re-extracted at a box size of 384 (0.854 Å per pixel) and subjected to 3D auto refinement, CTF refinement and a second 3D auto refinement^64^. The particles were then Bayesian particle polished^65^ before another round of two 3D auto refinements and a CTF refinement. This final particle stack was then imported back into cryoSPARC for a final NU refinement that resulted in a 3.22-Å-resolution map (Supplementary Table 1). 3DFSC was used to calculate directional Fourier shell correlation analysis for the final map^66^.

To generate a starting model, we used ModelAngelo^67^ and supplied our final 3.22 Å map and sequence files for IFIT2 and IFIT3. This resulting model was then iteratively real-space refined using Phenix^68^ and manually adjusted in COOT^69^. After the final refinement, the model was checked for accuracy in COOT (Supplementary Table 1).

### Evolutionary analyses

For evolutionary analyses of primate and rodent IFIT2 and IFIT3, Uniprot reference protein sequences for human IFIT2, human IFIT3, mouse IFIT2 and mouse IFIT3 were used as a search query against NCBI’s non-redundant (NR) database using tBLASTn^70^. Searches were restricted to simian primates and the Muroidea superfamily of rodents respectively. For each species, the nucleotide sequence with the highest bit score was downloaded and aligned to the human or mouse ORF nucleotide sequence using MAFFT^71^ implemented in Geneious software (Dotmatics; geneious.com). Poorly aligning sequences or regions were removed from subsequent analyses. Accession numbers of final sequences used for analyses are provided in Supplementary Table 2. Using these aligned sequences, FUBAR^72^ was performed on Datamonkey.org using 50 grid points and a 0.5 concentration parameter of the Dirichlet prior to infer codons evolving under positive and negative selection. Codons with a posterior probability of 0.9 or higher are given in Supplementary Table 2. PAML^73^ was used to infer gene-wide positive selection, as well as codon-based estimates of positive selection. Aligned sequences were analysed using the NS sites models, disallowing (M7) or allowing (M8) positive selection. The *P* value reported is the result of a chi-squared test on twice the difference of the log likelihood (lnL) values between the two models using two degrees of freedom. Analyses were performed using two models of frequency (F61 and F3×4), and both sets of values are reported in Supplementary Table 2. For each codon model, we confirmed convergence of lnL values by performing each analysis using two starting omega (d*N*/d*S*) values (0.4 and 1.5). Positively selected codons with a posterior probability >0.90 using a Bayes empirical Bayes (BEB) analysis and the F61 codon frequency model are provided in Supplementary Table 2.

### eCLIP experimental methods

Flp-In T-REx HEK293 cells in 10-cm culture dishes were induced with doxycycline for 24 h. Cells were then infected at an MOI of 3.0, and dishes were crosslinked using a UV crosslinker (254 nm; CL-1000 from UVP/Analytik Jena) at 400 mJ cm^−2^. Downstream sample processing and eCLIP were performed as previously described^74^, using antibodies against FLAG (M2/F1804, Sigma) and HA (HA.11/901502, Biolegend) for 293T experiments, and IFIT2 (PA3-845, Thermo Fisher) and IFIT3 (ABF1048, Millipore) for MEF experiments. Most experiments were performed in biological duplicate, except for the uninfected 293T samples (which were single replicates).

### eCLIP computational analysis

Standard processing of eCLIP data was performed as previously described^74^, with mapping performed to a custom genome index that included both the VSV genome and either hg19 (for 293T experiments) or mm10 (for MEF experiments). Data (Fig. 3 and Extended Data Fig. 4) are plotted as normalized reads per million (RPM), where reads per million are normalized to density of reads mapped to viral and human genomes.

### Generation of *Ifit3a/b* DKO mice

Wild-type C57BL/6J were commercially obtained from Jackson Laboratories (Strain 000664). To generate *Ifit3-*deficient mice, single guide RNAs (sgRNAs) were designed to target exon two in *Ifit3a* and *Ifit3b*. Two sgRNAs were chosen that target conserved sequences between *Ifit3a* and *Ifit3b*: sgRNA-4, 5’-ATTTCACCTGGAATTTATTCNGG-3’; and sgRNA-30, 5’-AATGGCACTTCAGCTGTGGANGG-3’. Two additional sgRNAs were chosen that target only *Ifit3a* due to polymorphisms: sgRNA-21, 5’-AATTCGTCGACTGGTCACCTNGG-3’; and sgRNA-22, 5’-ATTCGTCGACTGGTCACCTGNGG-3’. The sgRNAs were selected on the basis of their low off-target profile. Guide RNAs and Cas9 protein were complexed and electroporated concurrently into C57BL/6J zygotes. Using this approach, we identified a mouse that had both *Ifit3a* and *Ifit3b* targeted (22-nt and 20-nt frameshift deletions, respectively), two mice in which only *Ifit3a* was targeted (2-nt and 119-nt frameshift deletions), and two mice in which only *Ifit3b* was targeted (1-nt and 2-nt frameshift insertions). After genotyping and two rounds of backcrossing, five founder lines (*Ifit3a* del22/*Ifit3b* del20, *Ifit3a* del2, *Ifit3a* del119, *Ifit3b* ins1 and *Ifit3b* ins2) were generated. The generation of gene-edited mice was accomplished with the aid of the Genome Engineering and iPSC Center, and the Department of Pathology Micro-Injection Core (Washington University School of Medicine).

### 5’ RACE

HEK293T cells were maintained as described above and subcultured into 6-well plates (Genesee) in 2 ml of complete media at 24 h before transfection. Cells were transiently transfected with 2,500 ng of total DNA and 7.5 μl of TransIT-X2 (Mirus Bio) following manufacturer protocol. At 24 h post transfection, cells were collected and pelleted; cell pellets were washed with 1× PBS, pelleted again, and supernatant was aspirated. RNA was extracted from pellets using the Takara Bio NucleoSpin RNA Plus kit following manufacturer protocol. Downstream processing of RNA was performed using the Takara Bio SMARTer RACE 5’/3’ kit according to manufacturer protocol.

### Fluorescent reporter assay

Cells (HEK293T or inducible Flp-In lines) were maintained as described above and subcultured into 24-well plates for transfection. Transfections were performed as described above except for the addition of 100 ng of an mCherry-expressing DNA plasmid, resulting in 600 ng of total DNA transfected along with 1.8 μl of TransIT-X2. At 24 h post transfection, cells were imaged using the BioTek Cytation 5 cell imaging multimode reader. Four images were taken at fixed positions in each well using a ×20 objective lens, with each condition in two replicates; both GFP and RFP images were collected for each well position. Non-transfected wells were imaged for use in background subtraction. Images were preprocessed in the BioTek Gen5 Image Prime 3.1 software package (v.3.1.06) using the default rolling ball algorithm settings, and mean fluorescence values for GFP and RFP were quantified using the Gen5 software before being exported to Microsoft Excel 2019 (v.2507).

### Image analyses

Normalized GFP intensity for each image was calculated in Microsoft Excel as follows: (experimental GFP signal−background GFP signal)/(experimental RFP signal−background RFP signal). Background GFP and RFP signals are the average of the quantified preprocessed values from 8 total images taken of non-transfected wells. Once the normalized GFP intensity was calculated for each image, we averaged the normalized GFP intensity of each set of four images (that is, for each well). Finally, we calculated the relative GFP intensity for each well by dividing the average normalized GFP intensity from each well by the average of the two IFIT-untreated wells, thus representing each data point as relative to 100%.

### RT–qPCR

HEK293T cells were maintained as described above in 24-well plates. Cells were collected in 1× PBS and pelleted at 500 × *g* for 1 min. RNA was then extracted using the New England BioLabs Monarch Total RNA Miniprep kit according to manufacturer protocol. RNA (100 ng) was then subjected to reverse transcription using the Applied Biosystems High-Capacity cDNA Reverse Transcription kit according to manufacturer protocol. The resulting cDNA was then used to conduct quantitative PCR on the Applied Biosystems StepOnePlus machine with gene-specific primers (GFP-F, 5’-CCGACCACTACCAGCAGAACAC-3’; GFP-R, 5’-GGACCATGTGATCGCGCTTCTC-3’; 18S-F, 5’- TCGCTCGCTCCTCTCCTACTTG-3’; 18S-R, 5’- GCTGACCGGGTTGGTTTTGATCTG-3’) and the Luna Universal qPCR Master Mix according to manufacturer protocol.

### Statistics and reproducibility

Statistical analyses and data visualization were performed using GraphPad Prism 10 (v.10.4.1). Tests were performed as indicated in figure legends. All error bars represent s.e.m. No statistical method was used to predetermine sample size, but our sample sizes are similar to those reported in previous publications^75,76^. No data were excluded from the analyses. The experiments were not randomized. Data distribution was assumed to be normal, but this was not formally tested. Data collection and analysis were not performed blind to the conditions of the experiments, and the investigators were not blinded to allocation during experiments and outcome assessment.

### Reporting summary

Further information on research design is available in the Nature Portfolio Reporting Summary linked to this article.

## Supplementary information

Supplementary Information Supplementary Table 1.

Reporting Summary

Supplementary Table 2 Excel file containing evolutionary analyses-related information, related to Fig. 2 and Extended Data Fig. 4. Individual tables contain accession numbers and species for sequences analysed, complete lists of codons determined to be evolving under purifying and positive selection, and parameters and statistics for whole-gene and subdomain analyses.

Supplementary Table 3 Excel file containing information on plasmids and primers. Spreadsheet containing all plasmids used in this study, along with a brief description and primers and/or gene fragments used for cloning.

## Source data

Source Data Fig. 1 Statistical source data.

Source Data Fig. 1 Unprocessed blots.

Source Data Fig. 3 Statistical source data.

Source Data Fig. 5 Statistical source data.

Source Data Fig. 5 Unprocessed blots.

Source Data Fig. 6 Statistical source data.

Source Data Extended Data Fig. 1 Statistical source data.

Source Data Extended Data Fig. 1 Unprocessed blots.

Source Data Extended Data Fig. 4 Statistical source data.

Source Data Extended Data Fig. 5 Statistical source data.

Source Data Extended Data Fig. 5 Unprocessed blots.

Source Data Extended Data Fig. 6 Statistical source data.

Source Data Extended Data Fig. 7 Statistical source data.

Source Data Extended Data Fig. 8 Statistical source data.

## Figures and Tables

**Extended Data Table 1:**
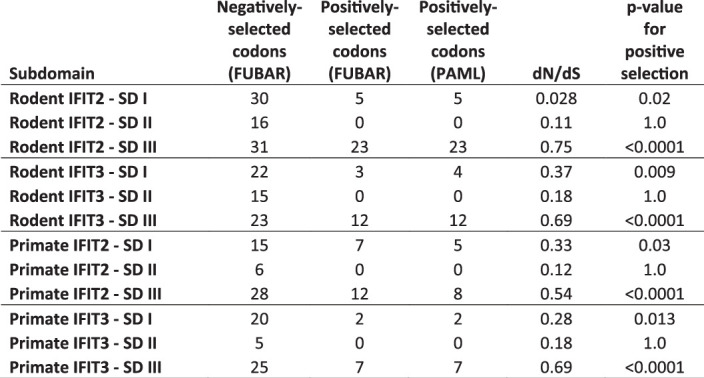
Evolutionary analysis, related to Fig. 2

Evolutionary analyses on subdomains of rodent and primate IFIT2 and IFIT3. Statistical evidence for positive selection, determined by PAML software, is shown for each gene and each subdomain, with statistically significant p-values shown in bold red text. The p-value reported is the result of a chi-squared test on twice the difference of the log likelihood (lnL) values between the two PAML models of selection (M7 and M8) using two degrees of freedom. Overall dN/dS (omega) values for each subdomain are also shown. The number of specific codons within each subdomain that show statistical evidence for positive and negative selection, as determined by PAML and FUBAR software as indicated. Additional statistics and analysis parameters, complete lists of codons, and accession numbers and species of sequences analyzed are found in Supplementary Table 2.

## Data Availability

All data reported in this paper are available in the paper, extended data, or as associated source data files (Source Data 1 contains datapoints shown in graphs, Source Data 2 contains all unedited western blot images). The cryo-EM structure has been deposited under PDB 9MK9 and EMDB code EMD-48323. Structural maps and model files are available through figshare at 10.6084/m9.figshare.28385627.v1 (ref. ^77^). Sequencing data from eCLIP experiment have been deposited in GEO record GSE284636. Source data are provided with this paper.

## Materials availability

All unique materials/reagents generated in this study are available through the lead contact (M.D.D., mddaugherty@ucsd.edu) upon request. Recipients are only expected to pay for shipping costs and there is no limitation on access. Every attempt will be made to send materials/reagents within one month of the request.
