# Supplementary material for: The IFIT2–IFIT3 antiviral complex targets short 5’ untranslated regions on viral mRNAs for translation inhibition
Source: Nat Microbiol. 2025 Oct 15;10(11):2934–48. doi: 10.1038/s41564-025-02138-w (PMC12570964; doi:10.1038/s41564-025-02138-w)
Supplement: Supplementary file 1 — Supplementary Table 1. [file 41564_2025_2138_MOESM1_ESM.pdf]

nature microbiology

Supplementary information https://doi.org/10.1038/s41564-025-02138-w

The IFIT2–IFIT3 antiviral complex targets short 5′ untranslated regions on viral mRNAs for translation inhibition

In the format provided by the authors and unedited

Supplementary Table 1. Cryo-electron microscopy data collection and structure determination, related to Fig. 2

**Data Collection**	
Magnification	165,000
Voltage (kV)	300
Movies	2355
Spherical Aberration (mm)	2.7
Electron Exposure ($e^-/Å^2$)	65
Defocus range (µm)	-1 to -2.5
Pixel size (Å, Physical/Digital)	0.854
Energy Filter Slit Width (eV)	10
**Map Statistics and Post-Processing**	
Symmetry imposed	C1
Map Resolution (Å)	3.22
Local resolution range for 75% of voxels ($Å^2$)	6.207
Local resolution range ($Å^2$)	2.9 - 26.6
Map sharpening B factor ($Å^2$)	107.7
Map sharpening method	B-Factor
Q-Score	0.58
**Model Statistics and Validation**	
Model composition	
Non-hydrogen atoms	6798
Protein residues	822
Water	47
R.M.S deviations	
Bond length (Å)	0.003
Bond angles (°)	0.592
MolProbity score	1.37
MolProbity Clashscore	6.62
CaBLAM (% outliers)	1.46
Rotamer outliers (%)	0.00
Ramachandran Plot	
Favored	97.67
Allowed	2.33
Outliers	0.0
