# Supplementary material for: The IFIT2–IFIT3 antiviral complex targets short 5’ untranslated regions on viral mRNAs for translation inhibition
Source: Nat Microbiol. 2025 Oct 15;10(11):2934–48. doi: 10.1038/s41564-025-02138-w (PMC12570964; doi:10.1038/s41564-025-02138-w)
Supplement: Supplementary file 2 — Reporting Summary [file 41564_2025_2138_MOESM2_ESM.pdf]

nature portfolio

Corresponding author(s): Matthew Daugherty

Last updated by author(s): Aug 18, 2025

Reporting Summary

Nature Portfolio wishes to improve the reproducibility of the work that we publish. This form provides structure for consistency and transparency in reporting. For further information on Nature Portfolio policies, see our Editorial Policies and the Editorial Policy Checklist.

Statistics

For all statistical analyses, confirm that the following items are present in the figure legend, table legend, main text, or Methods section.

n/a	Confirmed	
☐	☒	The exact sample size (*n*) for each experimental group/condition, given as a discrete number and unit of measurement
☐	☒	A statement on whether measurements were taken from distinct samples or whether the same sample was measured repeatedly
☐	☒	The statistical test(s) used AND whether they are one- or two-sided<br>*Only common tests should be described solely by name; describe more complex techniques in the Methods section.*
☐	☒	A description of all covariates tested
☐	☒	A description of any assumptions or corrections, such as tests of normality and adjustment for multiple comparisons
☐	☒	A full description of the statistical parameters including central tendency (e.g. means) or other basic estimates (e.g. regression coefficient) AND variation (e.g. standard deviation) or associated estimates of uncertainty (e.g. confidence intervals)
☐	☒	For null hypothesis testing, the test statistic (e.g. *F*, *t*, *r*) with confidence intervals, effect sizes, degrees of freedom and *P* value noted<br>*Give P values as exact values whenever suitable.*
☒	☐	For Bayesian analysis, information on the choice of priors and Markov chain Monte Carlo settings
☒	☐	For hierarchical and complex designs, identification of the appropriate level for tests and full reporting of outcomes
☒	☐	Estimates of effect sizes (e.g. Cohen's *d*, Pearson's *r*), indicating how they were calculated

Our web collection on statistics for biologists contains articles on many of the points above.

Software and code

Policy information about availability of computer code

Data collection	Fluorescent images were captured, preprocessed, and quantified using the BioTek Gen5 Image Prime 3.1 Software for Imaging & Microscopy version 3.1.06. Western blots were imaged using the Bio-Rad Image Lab software version 6.1.0. qRT-PCR data were collected using the Applied Biosystems StepOnePlus machine.
Data analysis	Data analysis for virological assays and reporter assays was conducted using Microsoft Excel 2019 version 2507 and GraphPad Prism 10 version 10.4.1. Evolutionary analyses were performed with PAML (version 4.9), MAFFT (version 7.388), Geneious (version 2025.0.1), and FUBAR (Datamonkey online version),

For manuscripts utilizing custom algorithms or software that are central to the research but not yet described in published literature, software must be made available to editors and reviewers. We strongly encourage code deposition in a community repository (e.g. GitHub). See the Nature Portfolio guidelines for submitting code & software for further information.

Data

Policy information about availability of data

All manuscripts must include a data availability statement. This statement should provide the following information, where applicable:

- Accession codes, unique identifiers, or web links for publicly available datasets
- A description of any restrictions on data availability
- For clinical datasets or third party data, please ensure that the statement adheres to our policy

All data reported in this paper are available in the manuscript, extended data, or as associated source data files (Source Data 1 contains datapoints shown in graphs, Source Data 2 contains all unedited western blot images). The cryo-EM structure has been deposited under PDB 9MK9 and EMDB code EMD-48323. Structural maps and model files are available through figshare at https://doi.org/10.6084/m9.figshare.28385627.v1. Sequencing data from eCLIP experiment have been deposited in GEO record GSE284636.

Research involving human participants, their data, or biological material

Policy information about studies with human participants or human data. See also policy information about sex, gender (identity/presentation), and sexual orientation and race, ethnicity and racism.

Reporting on sex and gender	*Use the terms sex (biological attribute) and gender (shaped by social and cultural circumstances) carefully in order to avoid confusing both terms. Indicate if findings apply to only one sex or gender; describe whether sex and gender were considered in study design; whether sex and/or gender was determined based on self-reporting or assigned and methods used. Provide in the source data disaggregated sex and gender data, where this information has been collected, and if consent has been obtained for sharing of individual-level data; provide overall numbers in this Reporting Summary. Please state if this information has not been collected. Report sex- and gender-based analyses where performed, justify reasons for lack of sex- and gender-based analysis.*
Reporting on race, ethnicity, or other socially relevant groupings	*Please specify the socially constructed or socially relevant categorization variable(s) used in your manuscript and explain why they were used. Please note that such variables should not be used as proxies for other socially constructed/relevant variables (for example, race or ethnicity should not be used as a proxy for socioeconomic status). Provide clear definitions of the relevant terms used, how they were provided (by the participants/respondents, the researchers, or third parties), and the method(s) used to classify people into the different categories (e.g. self-report, census or administrative data, social media data, etc.) Please provide details about how you controlled for confounding variables in your analyses.*
Population characteristics	*Describe the covariate-relevant population characteristics of the human research participants (e.g. age, genotypic information, past and current diagnosis and treatment categories). If you filled out the behavioural & social sciences study design questions and have nothing to add here, write "See above."*
Recruitment	*Describe how participants were recruited. Outline any potential self-selection bias or other biases that may be present and how these are likely to impact results.*
Ethics oversight	*Identify the organization(s) that approved the study protocol.*

Note that full information on the approval of the study protocol must also be provided in the manuscript.

Field-specific reporting

Please select the one below that is the best fit for your research. If you are not sure, read the appropriate sections before making your selection.

☒ Life sciences ☐ Behavioural & social sciences ☐ Ecological, evolutionary & environmental sciences

For a reference copy of the document with all sections, see nature.com/documents/nr-reporting-summary-flat.pdf

Life sciences study design

All studies must disclose on these points even when the disclosure is negative.

Sample size	No statistical method was used to predetermine sample size but our sample sizes are similar to those reported in previous publications (Tsu et al. 2003. PLoS Biol; Ryan et al. 2025. Sci Adv).
Data exclusions	No data were excluded from these analyses.
Replication	Experiments were performed multiple independent times, often by different researchers. All replication attempts were successful. Experiments were performed with a minimum of biological duplicate conditions, and each experiment was independently performed at least twice (for a total of at least 4 biological replicates).
Randomization	Experiments were not randomized, as this was not relevant to our study. Experiments were performed in the same cell line, where the tested conditions were -/+ viral infection, -/+ a reporter plasmid, and/or -/+ treatment with IFIT, interferon, or doxycycline.

Blinding	Blinding was not relevant to the study, as the performed experiments all produced quantitative data that were not open to interpretation by different researchers.

Reporting for specific materials, systems and methods

We require information from authors about some types of materials, experimental systems and methods used in many studies. Here, indicate whether each material, system or method listed is relevant to your study. If you are not sure if a list item applies to your research, read the appropriate section before selecting a response.

Materials & experimental systems

n/a	Involved in the study
☐	☒ Antibodies
☐	☒ Eukaryotic cell lines
☒	☐ Palaeontology and archaeology
☒	☐ Animals and other organisms
☒	☐ Clinical data
☒	☐ Dual use research of concern
☒	☐ Plants

Methods

n/a	Involved in the study
☒	☐ ChIP-seq
☒	☐ Flow cytometry
☒	☐ MRI-based neuroimaging

Antibodies

Antibodies used

Primary Antibodies (all used at 1:1000 dilution): anti-VSV-G [8G5F11], anti-VSV-N [10G4] (Kerafast); anti-V5 [D3H8Q] (Cell Signaling Technology); anti-GAPDH [14C10] (Cell Signaling Technology); HA [3F10] (Roche); FLAG [M2] (Sigma); HaloTag (Promega); human IFIT1 [3G8] (Novus Biologicals)

Secondary Antibodies (all used at 1:10000 dilution): Goat Anti-Rabbit IgG (H + L)-HRP Conjugate (Bio-Rad), Goat Anti-Mouse IgG (H + L)-HRP Conjugate (Bio-Rad), Goat anti-Rat IgG (H+L) Secondary Antibody, HRP (Invitrogen)

Validation

anti-VSV-G: from the Kerafast website - "BHK cells were infected with VSV and labeled with Anti-VSV-G (8G5F11) and JacksonLabs Dylight 549 goat anti-mouse secondary antibody. Cells were imaged at 20X using QImaging Camera (half of 14-bit range with < 1000 ms exposure). Cells were stained and imaged live. (contrast adjusted 8-bit image)." There are also 46 related publications listed.

Anti VSV-N: the Kerafast website lists 9 related publications.

Anti-V5: the Cell Signaling Technology website presents validation data from western blots, immunoprecipitation, microscopy, and flow cytometry. There are 464 related publications.

Anti-GAPDH: the Cell Signaling Technology website presents validation data from western blots, immunohistochemistry, microscopy, and flow cytometry. There are 8933 related publications.

Anti-HA: the Sigma website presents validation data from western blots. There are 41 related publications.

Anti-FLAG: the Sigma website presents validation data from microscopy, western blots, and ELISA. There are 9676 related publications.

Anti-HaloTag: the Promega website presents validation data from western blots. They do not list related publications.

Anti-Human IFIT1: the Novus website presents validation data from western blots, immunohistochemistry, microscopy, and flow cytometry. There is 1 related publication.

Goat Anti-Rabbit IgG (H + L)-HRP Conjugate, Goat Anti-Mouse IgG (H + L)-HRP Conjugate: Bio-Rad provides a PDF of supporting documentation that lists 1 related publication.

Goat anti-Rat IgG (H+L) Secondary Antibody, HRP (Invitrogen) : the Invitrogen website provides multiple figures, including blots, of supporting data for validation.

Eukaryotic cell lines

Policy information about cell lines and Sex and Gender in Research

Cell line source(s)

HEK293T, BHK-21, and H1HeLa cells were obtained from ATCC (catalog # CRL-3216; # CCL-10; # CRL-1958, respectively).
Flp-In T-REx HEK293 cells were obtained from Thermo Fisher (Invitrogen, catalog # R78007). Inducible lines expressing mCherry, IFIT2, IFIT3, and IFIT2-IFIT3 were generated according to the manufacturer's protocol.
MEFs from Ifit2-/- and ΔIfit3a/b mice were prepared from day 13.5-14.5 embryos according to published protocols (Tan Y.S. and Lei Y.L., 2019. Methods Mol Biol.). Because of the early stage of embryo development, mice could not be externally sexed; however, since gross decreases in litter size for the KO animals (8-10 pups per litter) were not observed, we assume that there was a mix of male and female embryos.

Authentication	HEK293 cells were authenticated by STR. Successful generation of inducible Flp-In lines were authenticated by induction with doxycycline followed by fluorescence imaging for mCherry or western blot for IFIT2 and/or IFIT3.
Mycoplasma contamination	All lines are regularly tested for Mycoplasma contamination, and cell lines used in this manuscript were Mycoplasma-free.
Commonly misidentified lines (See ICLAC register)	None of our cell lines appear on the list.

Plants

Seed stocks	*Report on the source of all seed stocks or other plant material used. If applicable, state the seed stock centre and catalogue number. If plant specimens were collected from the field, describe the collection location, date and sampling procedures.*
Novel plant genotypes	*Describe the methods by which all novel plant genotypes were produced. This includes those generated by transgenic approaches, gene editing, chemical/radiation-based mutagenesis and hybridization. For transgenic lines, describe the transformation method, the number of independent lines analyzed and the generation upon which experiments were performed. For gene-edited lines, describe the editor used, the endogenous sequence targeted for editing, the targeting guide RNA sequence (if applicable) and how the editor was applied.*
Authentication	*Describe any authentication procedures for each seed stock used or novel genotype generated. Describe any experiments used to assess the effect of a mutation and, where applicable, how potential secondary effects (e.g. second site T-DNA insertions, mosiacism, off-target gene editing) were examined.*
