# Supplementary figures and images for: The IFIT2–IFIT3 antiviral complex targets short 5’ untranslated regions on viral mRNAs for translation inhibition
Source: Nat Microbiol. 2025 Oct 15;10(11):2934–48. doi: 10.1038/s41564-025-02138-w (PMC12570964; doi:10.1038/s41564-025-02138-w)

**e** $\alpha$ -VSV-G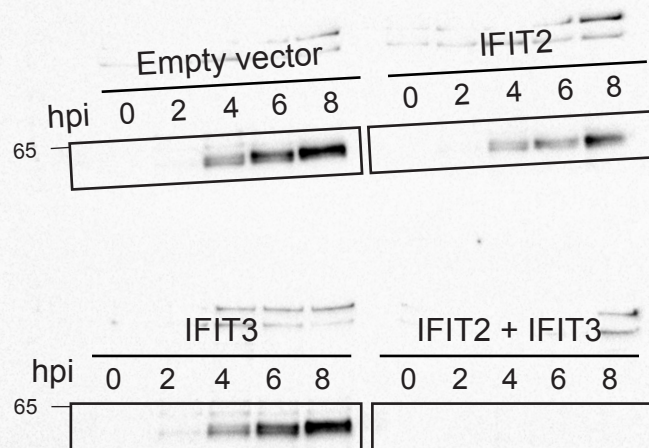 $\alpha$ -VSV-N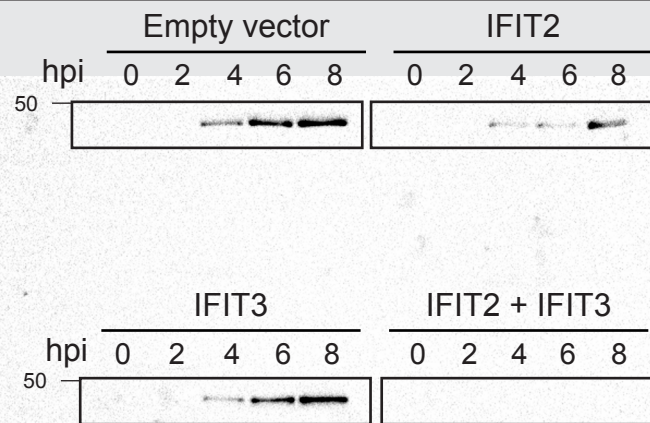 $\alpha$ -GAPDH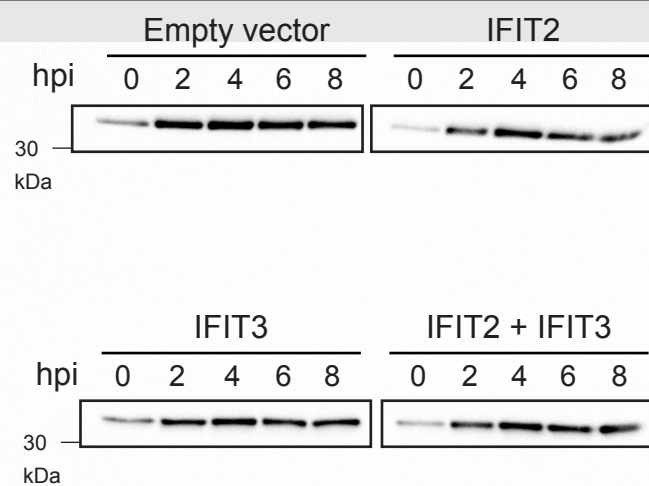

Supplement: Supplementary file 6 — Unprocessed blots. [file 41564_2025_2138_MOESM6_ESM.pdf]

**a**

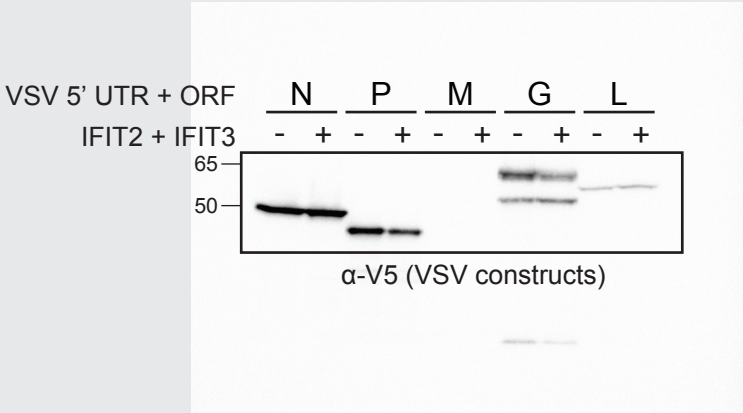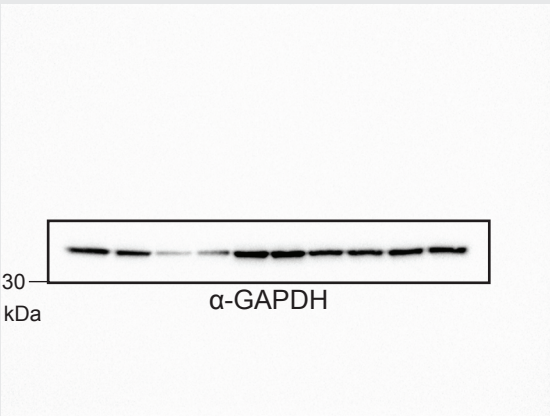

**b**

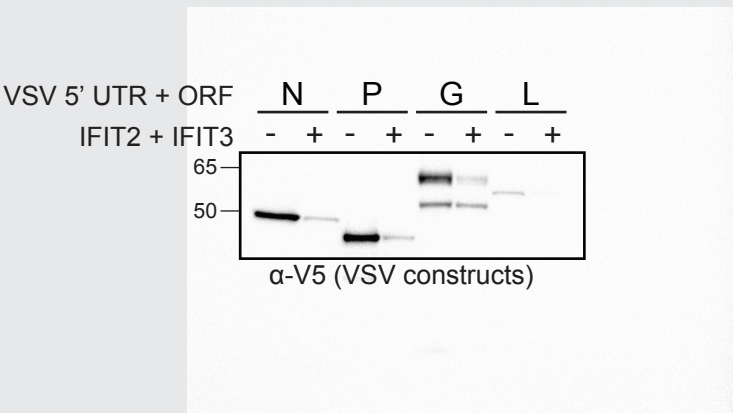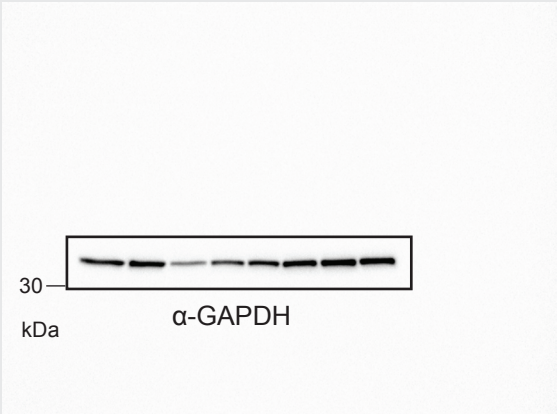

Supplement: Supplementary file 9 — Unprocessed blots. [file 41564_2025_2138_MOESM9_ESM.pdf]

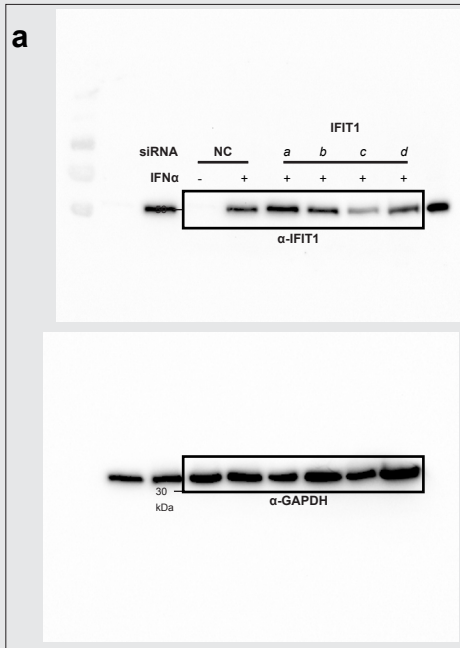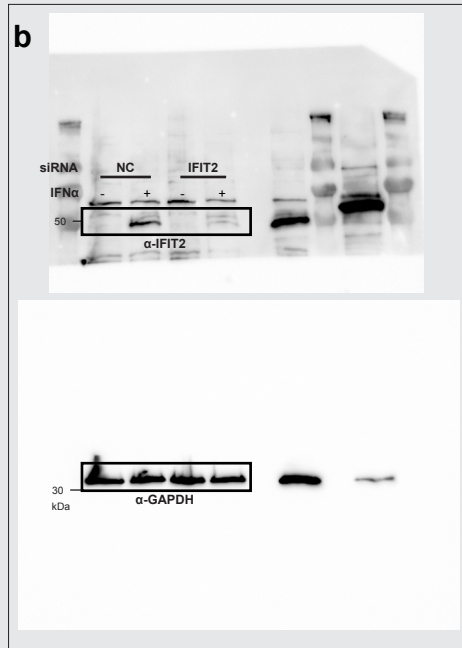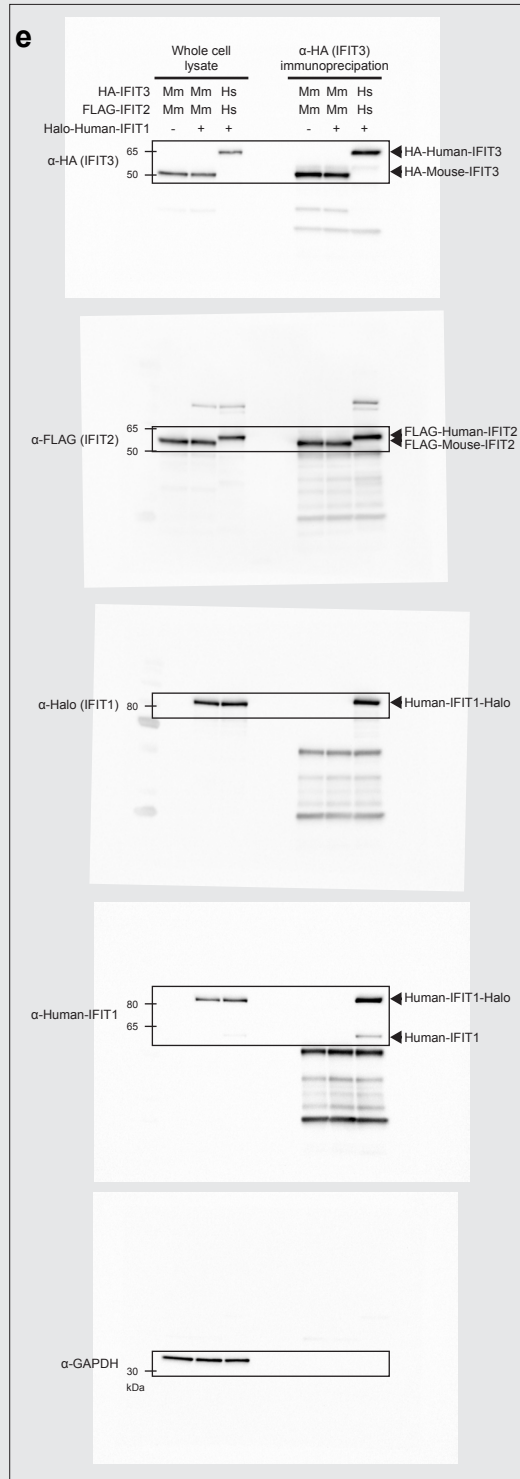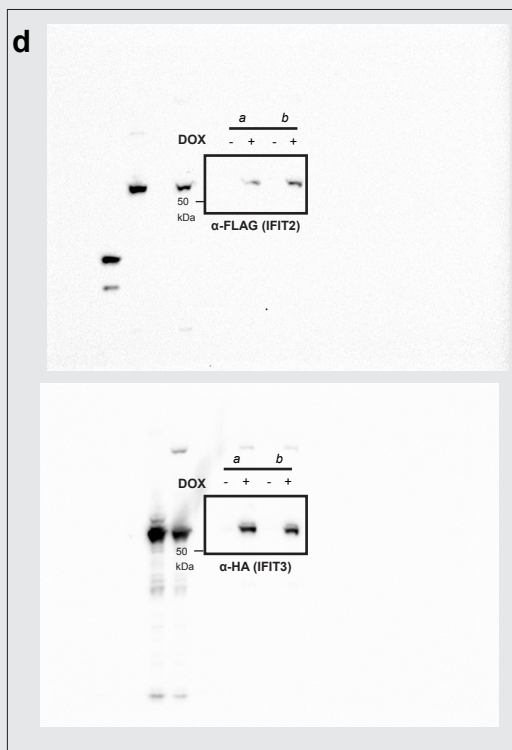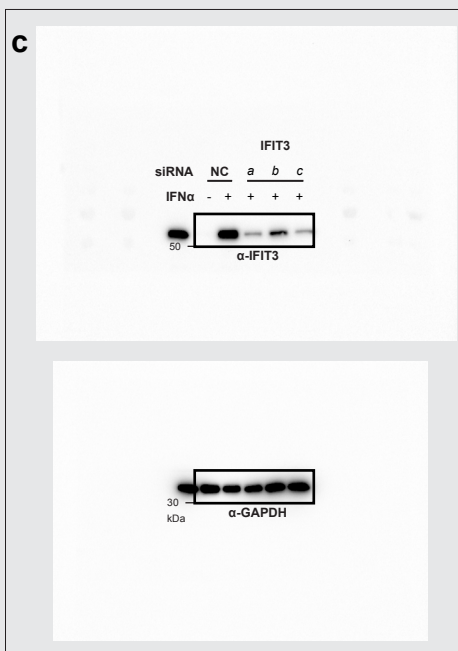

Supplement: Supplementary file 12 — Unprocessed blots. [file 41564_2025_2138_MOESM12_ESM.pdf]

**C**

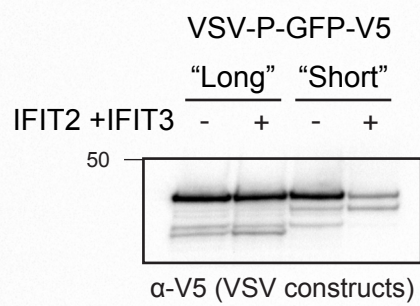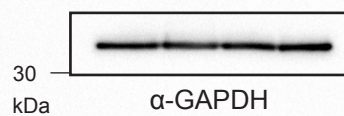

Supplement: Supplementary file 15 — Unprocessed blots. [file 41564_2025_2138_MOESM15_ESM.pdf]
